# Supplementary figures and images for: A genome-wide comprehensive analysis of nucleosome positioning in yeast
Source: PLoS Comput Biol. 2024 Jan 24;20(1):e1011799. doi: 10.1371/journal.pcbi.1011799 (PMC10843174; doi:10.1371/journal.pcbi.1011799)

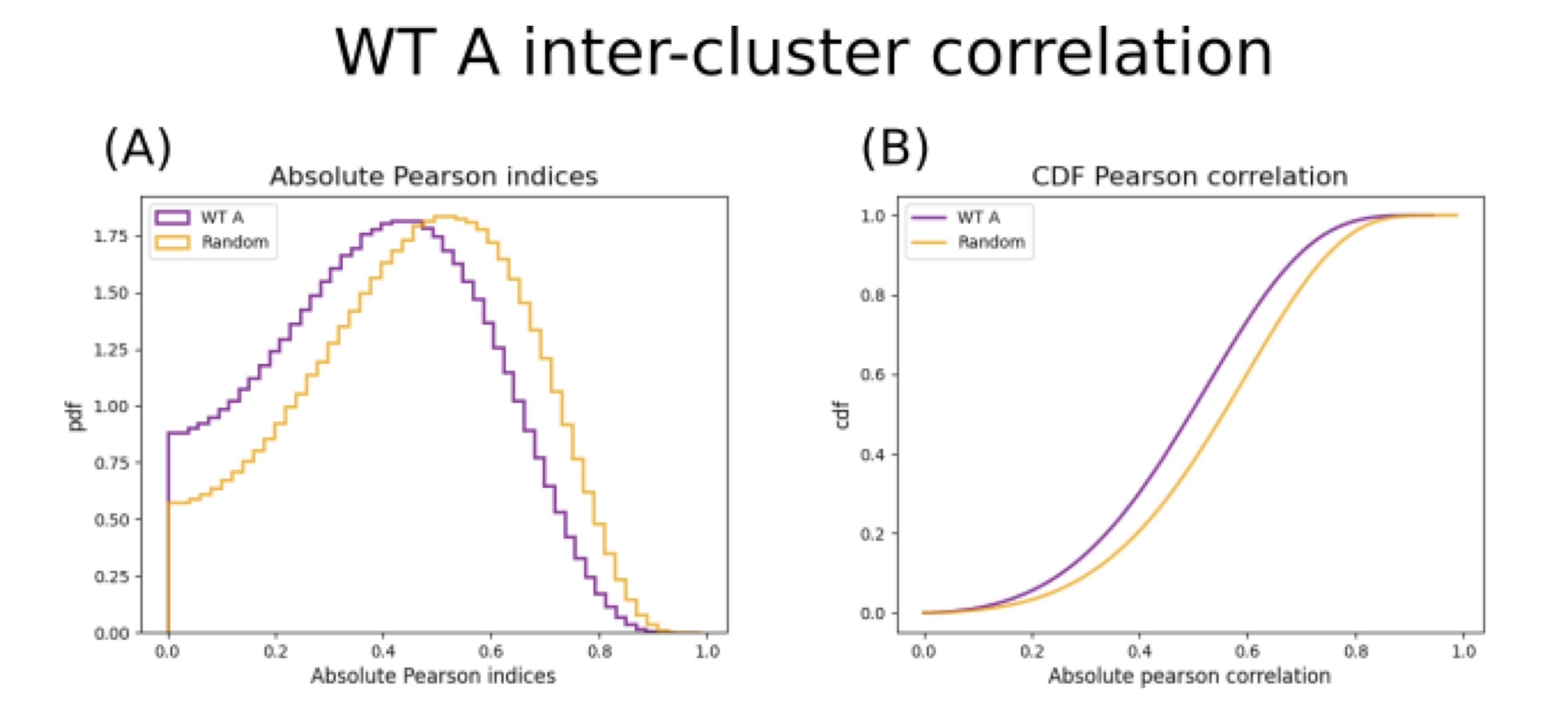

Supplement: S1 Fig — We measure the inter-cluster correlation for the found clusters and 500 random clusters. It is expected that the found gene groups have a significantly lower inter-cluster correlation than a random separation (i.e. the found clusters are most dissimilar based on the Pearson correlation). A KS test over 500 randomly sampled inter-cluster correlation indices for the k-mean and random grouping, respectively, proved their significance. Magenta shows the distribution for the found clusters, orange displays one example for random clustering. (A) The distribution over the absolute pairwise inter-cluster correlations is significantly lower for the gene groups determined by the k-mean clustering than for the random grouping. (B) The cumulative distribution function for the found clusters raises much more quickly. A KS test verified that this trend is indeed significant (average p-value over 500 repetitions 0.0009). (TIFF) [file pcbi.1011799.s001.tiff]

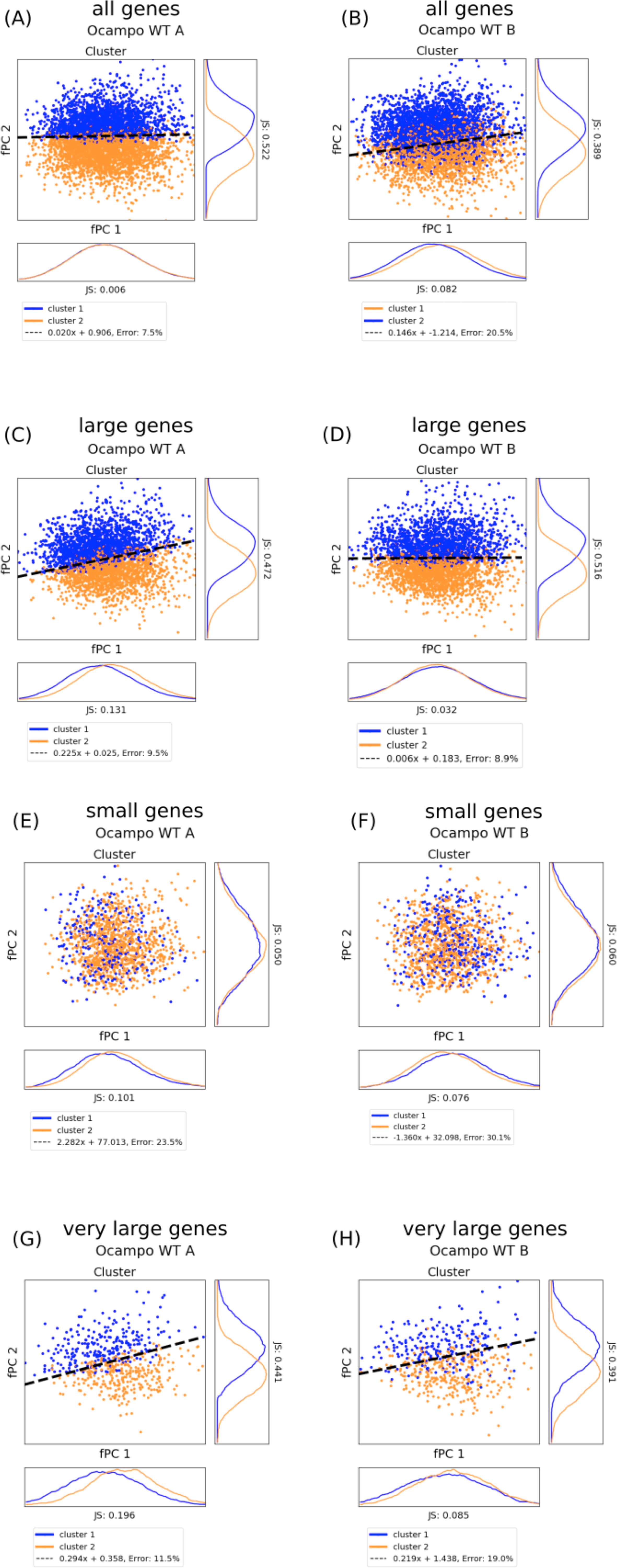

Supplement: S2 Fig — Blue and orange indicate each one group, the dashed line symbolises the best linear separation using a SVM. The x-axis represents the score of the first fPC ζ1, the y-axis gives the score for the second fPC ζ2. All axes are scaled to the same size; shapes are therefore comparable. (A) and (B) show all genes for replicate A and B. (C) and (D) display the fPC scores after filtering for large genes (>1000 bp) for replicates A and B. (E) and (F) show small genes ((≤1000 bp) for replicate A and B. We removed the separating boundary because it did not reasonably divide the clusters. Nevertheless, we kept the estimated linear function in the legend to allow a comparison with other boundaries. Of particular note is the bias, which can be even order of magnitudes different from large-gene clusters. (G) and (H) display the fPC scores after filtering for very large genes (>3000 bp) for replicates A and B. (TIFF) [file pcbi.1011799.s002.tiff]

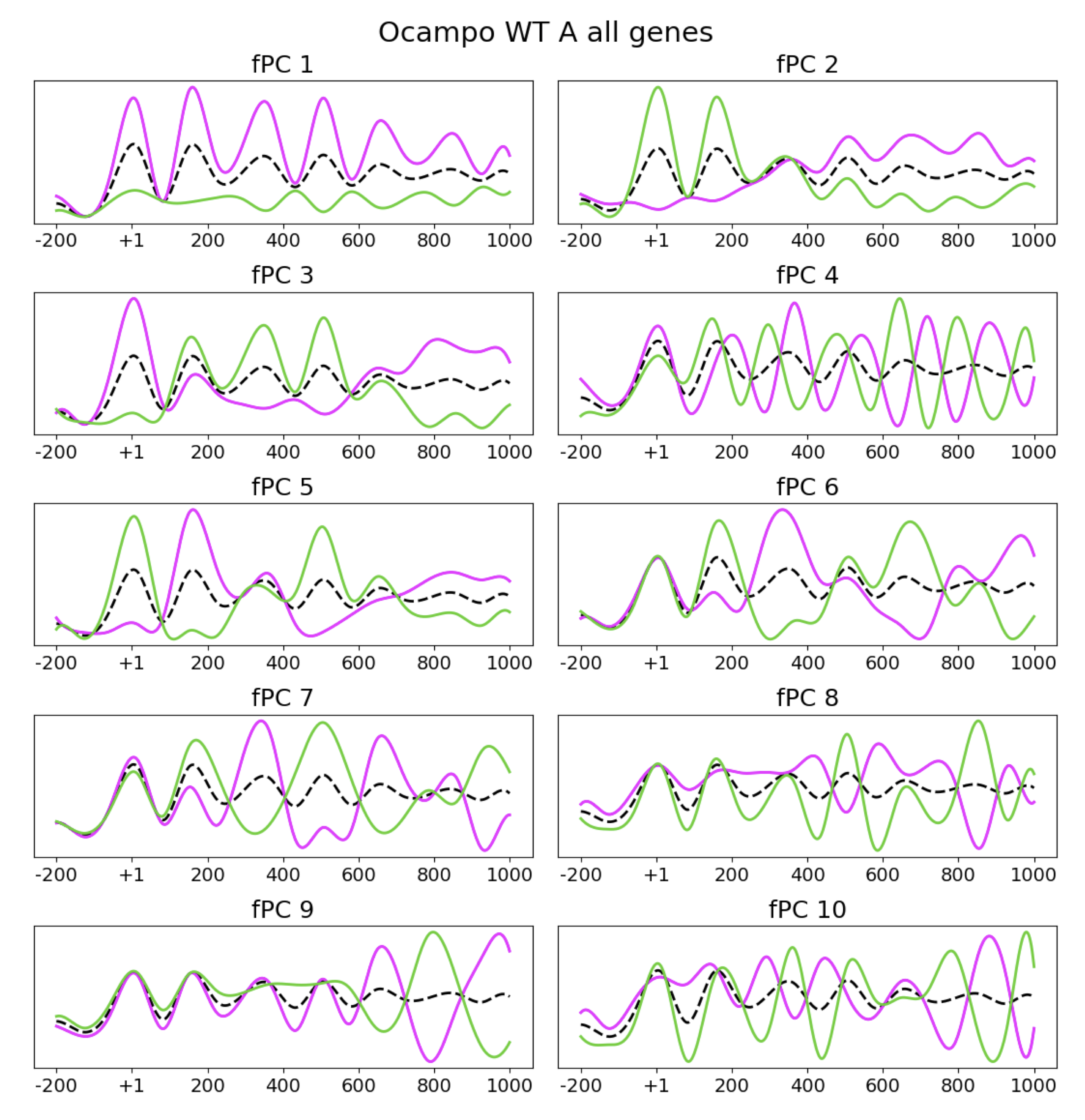

Supplement: S3 Fig — FPCs are ordered with respect to how much variance they explain (i.e. fPC1 explains the most whereas fPC10 explains the least). The mean is given as a black dashed line, a positive contribution is shown in magenta, whereas a negative contribution is displayed in green. The first two fPCs are the ones that were presented in Fig 1(F) (21.3% and 11.5% explained variance, respectively). The fPCs that follow after the major two ones become increasingly complex, and it is difficult to quantify their effect in a straightforward measurement. With the exception of fPC4 (7.8% explained variance), the plots suggest that the effects of the fPCs that were not included would not have been captured by the linear correlation index, as they describe changes specific to a single nucleosome (e.g. fPC6) or complex changes (e.g. fPC7). We want to remind that the variance captured by the fPCs could either moderately occur along the majority of genes; or alternatively, there is a strong effect on a small subset of profiles. We interpret the results as follows. FPC1 and fPC2 show global trends how nucleosome arrangements change along most protein-coding regions. This is emphasised by the fact that they capture the most deviance from the mean and that their effects are not specific to particular nucleosome positions. Many other fPCs (e.g fPCs 5, 6, and 7, 7.5%, 7%, and 6.1% explained variance, respectively) include strong position-specific effects. Despite the large impact on the amplitude at precise positions (e.g. +3 or +4), the explained variance by these fPCs is lower. We presume that this indicates a strong variance at these positions in a small subset of genes. Thus, they show a much lower variance. We focus on the first two fPCs because they can separate the Pearson clusters, which in turn indicates that they capture global trends since they were determined over all genes. (TIFF) [file pcbi.1011799.s003.tiff]

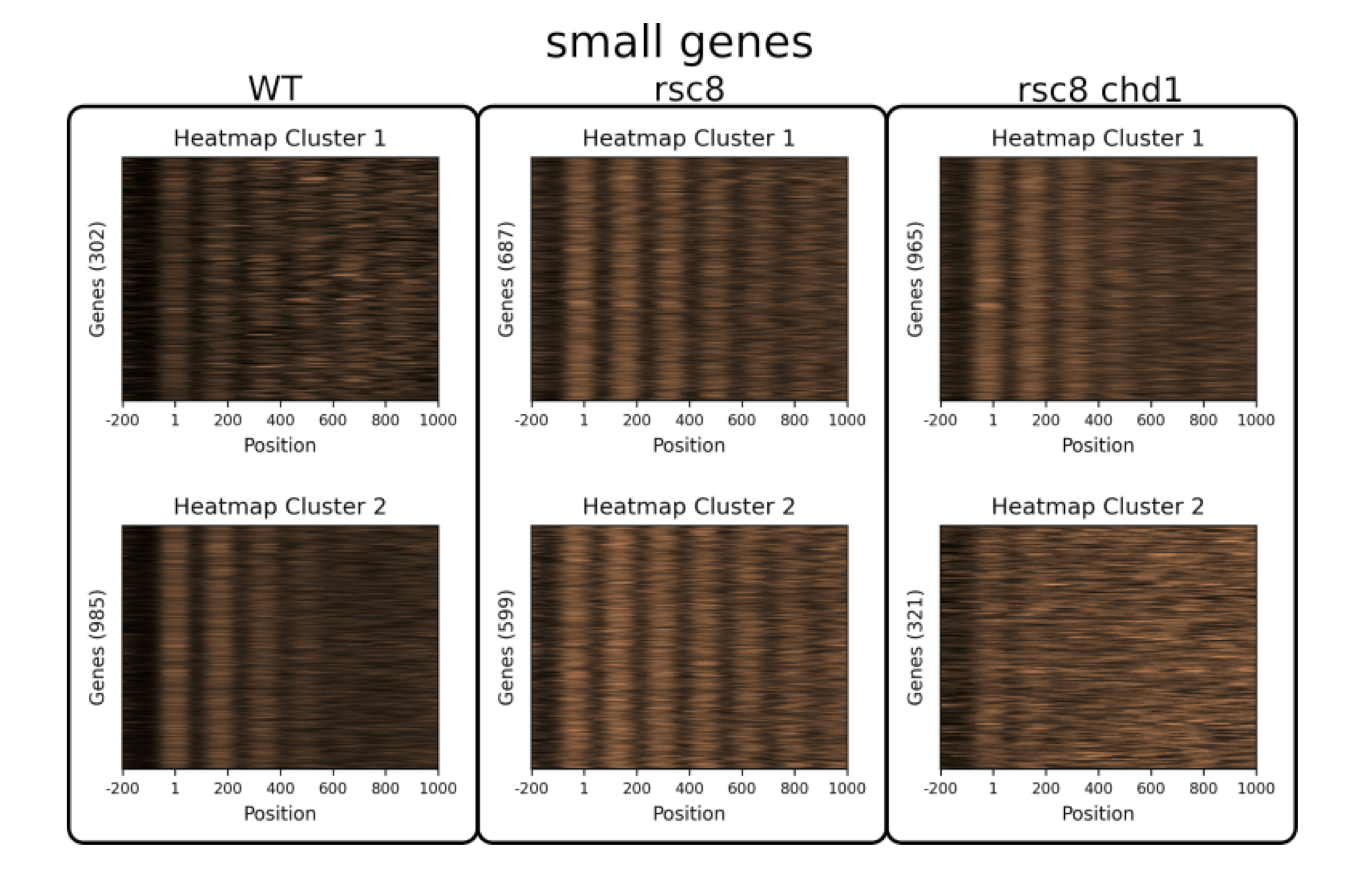

Supplement: S4 Fig — Cluster 1 and 2 for all genes in WT conditions were plotted only including small genes on the left. Indeed, correct positioning is either completely disrupted (Cluster 1), or clear phasing is lost after +3 or +4 position and individual peaks do not stand out thereafter (Cluster 2). However, both Pearson clusters for rsc8-depleted cells (centre) show clear phasing probabilities, despite all genes being smaller than the considered 1000 bp after the +1. The double mutant chd1Δrsc8 seems to re-establish the gene boundaries for nucleosome phasing, as positioning is either disrupted (Cluster 2, compare with Cluster 1 in WT) or does not exhibit clearly distinguishable peaks after the +3 or +4 nucleosome (Cluster 1, compare with Cluster 2 in WT). Defining a group as being 1 or 2 was arbitrary and has no significance. Copper values show large MNase-seq signal values, whereas dark segments indicate a low amplitude. Values in between are uniformly scaled. (TIFF) [file pcbi.1011799.s004.tiff]

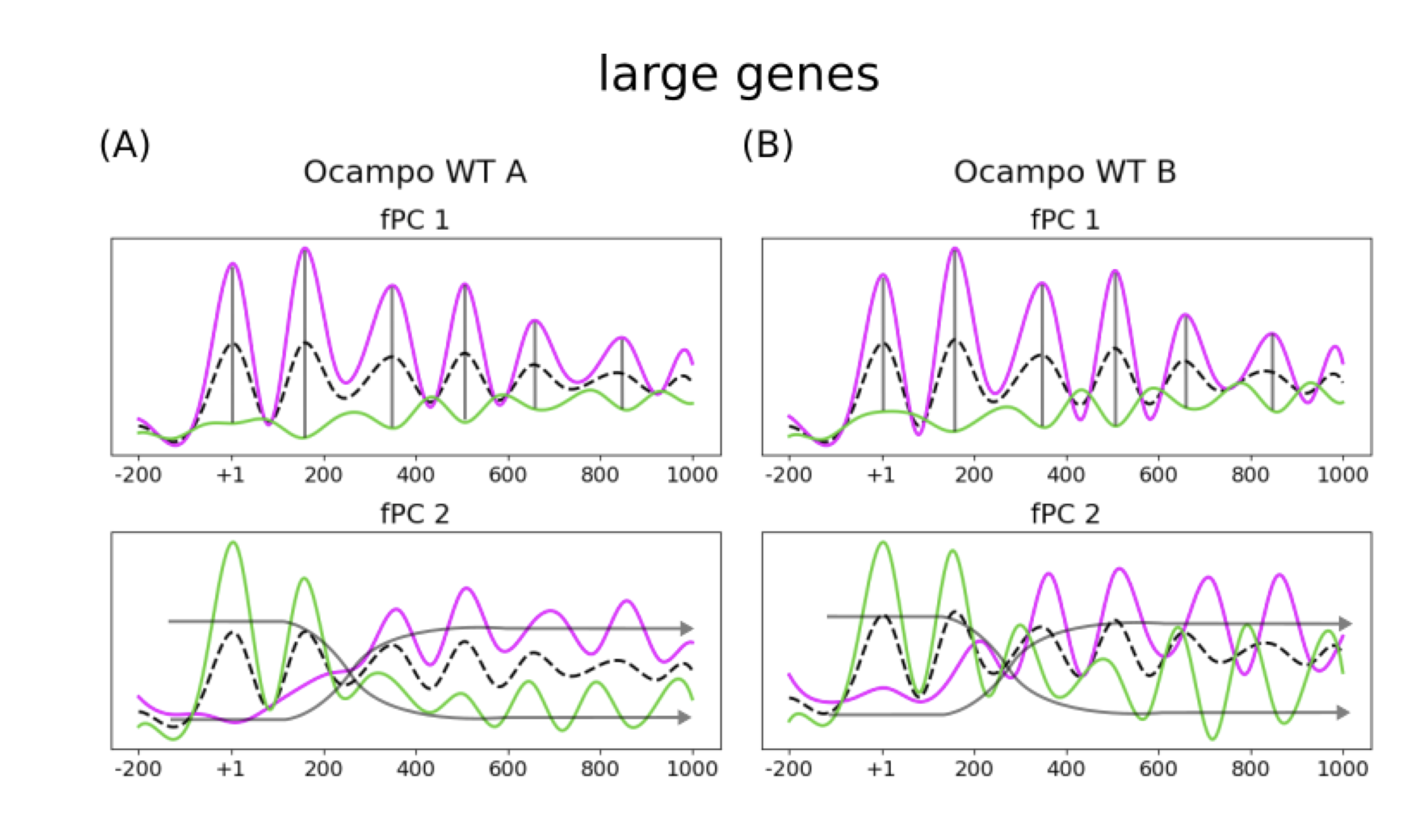

Supplement: S5 Fig — Despite fact that the functions differ in the A and B replicate ((A) and (B)), they both describe the same properties as when considering all genes (Fig 1(F)). To be precise, the first fPC describes seemingly position-dependent scaling (grey vertical bars), and the second explains coordinated phasing (grey arrows). The mean is displayed as a black dashed line, whereas a positive and a negative functional contribution are given in magenta and green, respectively. (TIFF) [file pcbi.1011799.s005.tiff]

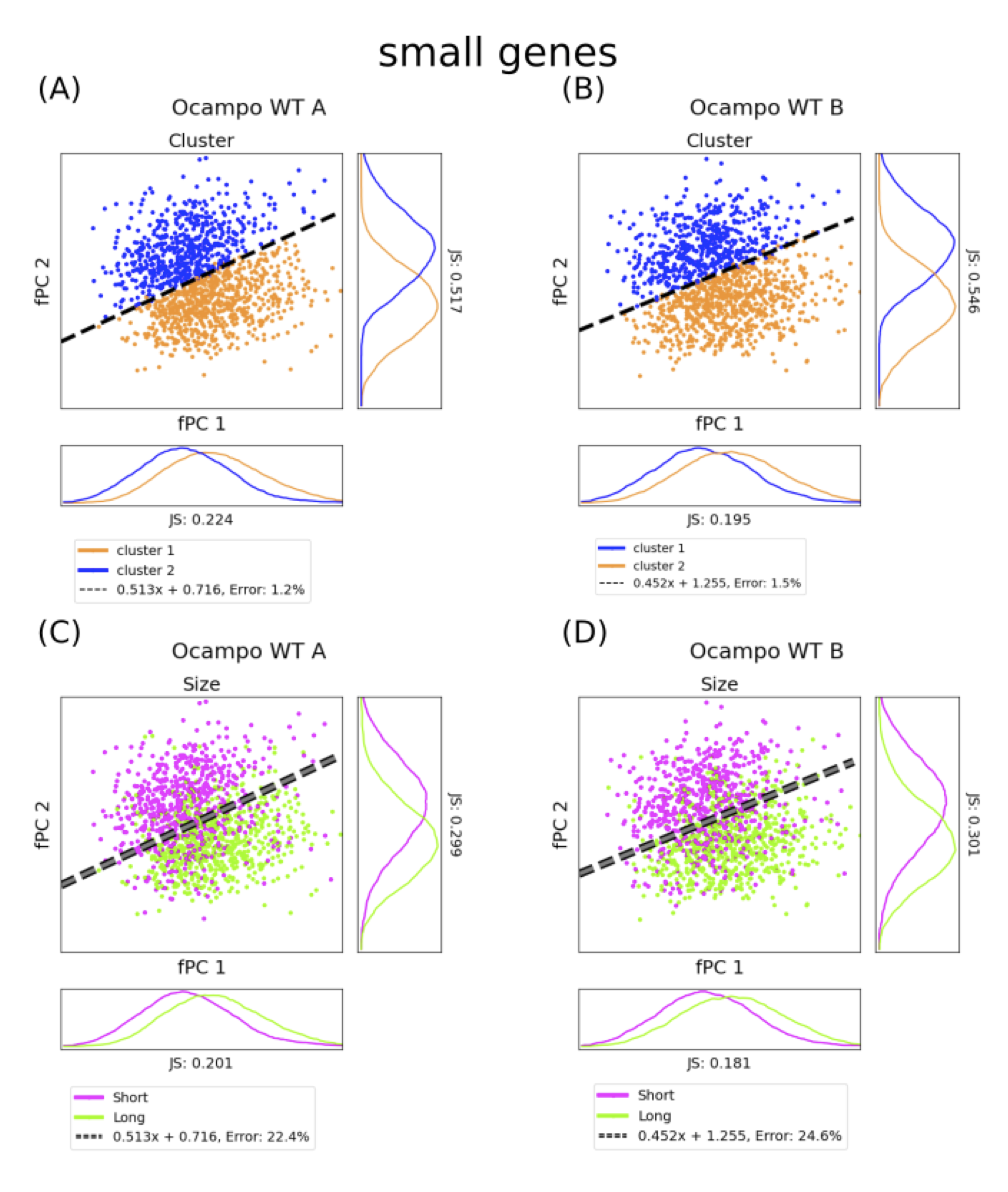

Supplement: S6 Fig — When we repeated the Pearson coefficient clustering considering exclusively small genes, we can linearly separate again the two groups (orange and blue). However, this is predominantly explained by the size of the gene (short pink, long green). This in line with the hypothesis that coordinated nucleosome phasing along the transcribed region is strictly limited within the gene body. The phase separating line was determined on the Pearson clusters (dashed black line) using an SVM. The same separating boundary was also plotted in right plot showing grouping with respect to the size. We plotted the original SVM boundary from the Pearson clusters with a dashed grey line to indicate that it was not determined using gene size. (A) and (B) give the Pearson clusters for replicate A and B. (C) and (D) show the size dependence of replicate A and B. (TIFF) [file pcbi.1011799.s006.tiff]

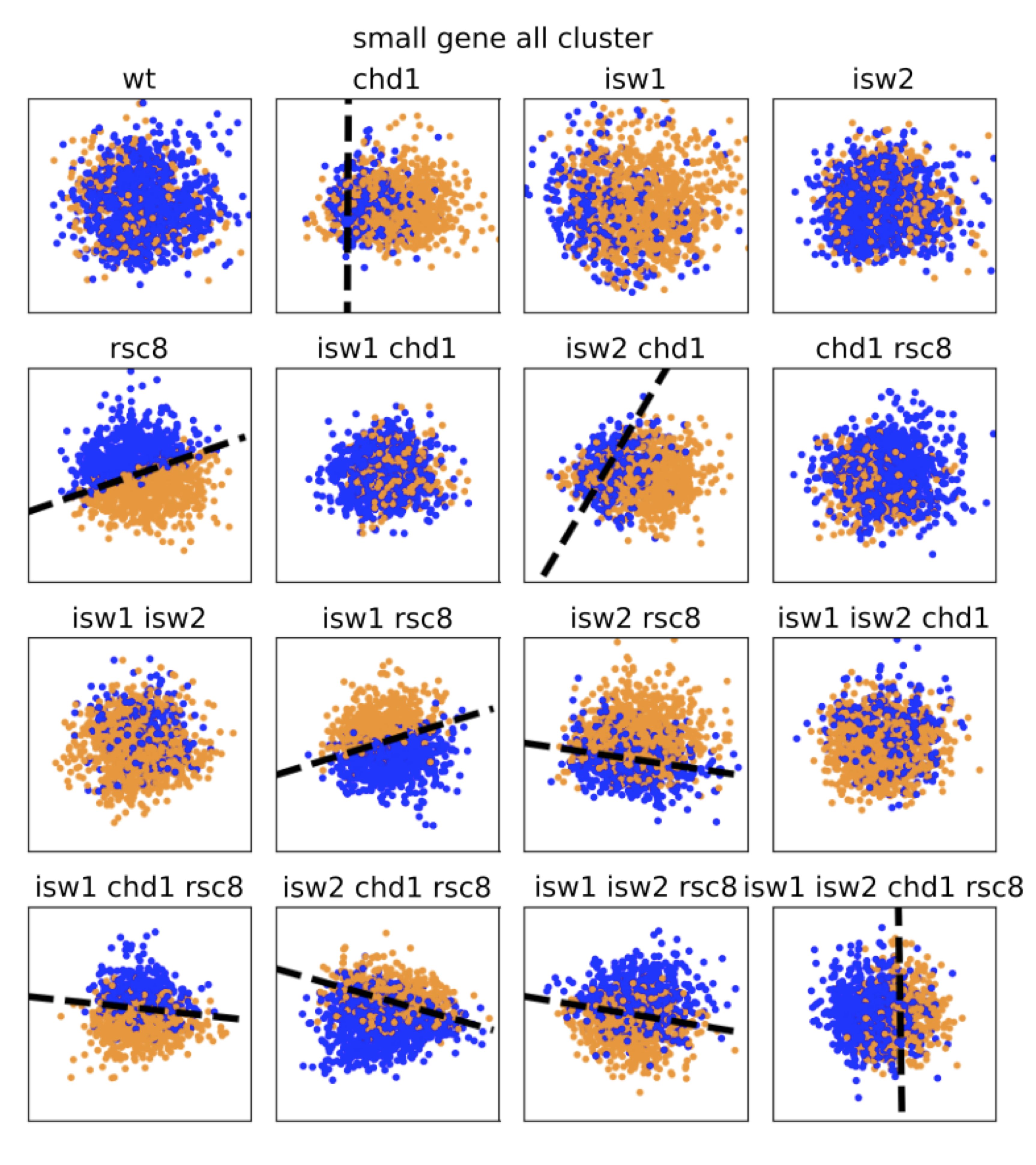

Supplement: S7 Fig — The figure shows the fPC scores ζ of small genes (<1000 bp) of all conditions coloured with respect to the all-gene Pearson clustering. Blue and orange indicate each one group, the dashed line symbolises the best linear separation using a SVM. We removed the linear boundary in plots where it went through the periphery instead of dividing the data points. The x-axis represents the score of the first fPC ζ1, the y-axis gives the score for the second fPC ζ2. All axes are scaled to the same size; shapes are therefore comparable. (TIFF) [file pcbi.1011799.s007.tiff]

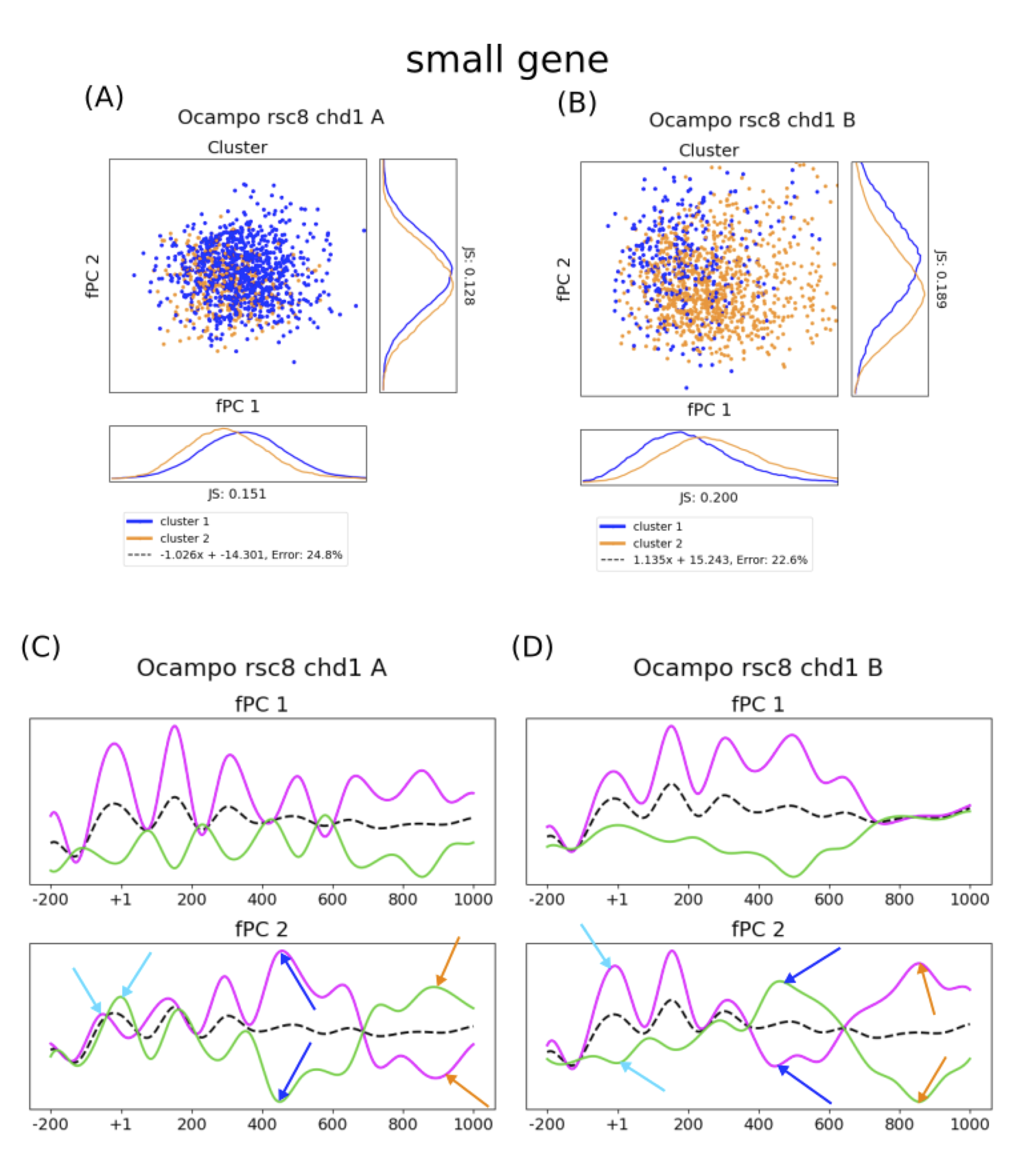

Supplement: S8 Fig — The double mutant seemingly re-establishes gene boundaries, and coordinated phasing is at least weakened after the +2 nucleosome (+1 in turquoise, +4 in blue, +6 in orange). This is true despite the fact that the A and B replicate differ. Figs (A) and (B) show the clusters for replicate A and B, and Figs (C) and (D) display their fPCs. We removed the separating boundaries in (A) and (B) because they did not reasonably divide the clusters. Nevertheless, we kept the estimated linear function in the legend to allow a comparison with other boundaries. Of particular note is the bias, which differs largely from large-gene clusters. The dashed black lines, the solid purple, and the solid green lines indicate the mean, a positive contribution, and a negative contribution, respectively. (TIFF) [file pcbi.1011799.s008.tiff]

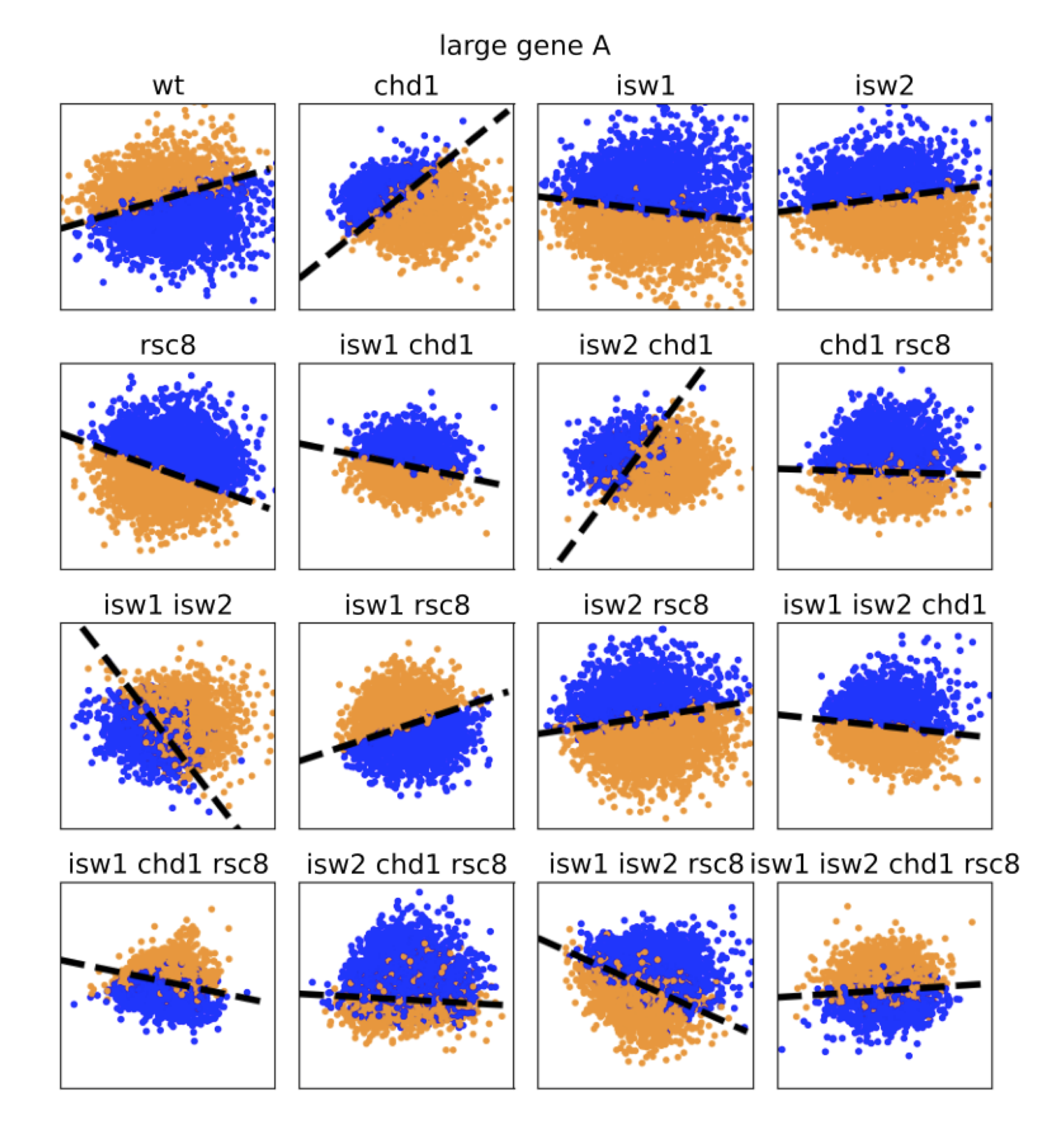

Supplement: S10 Fig — The figure shows the fPC scores ζ of all conditions coloured with respect to the Pearson clustering using only large genes (≥1000 bp). Blue and orange indicate each one group, the dashed line symbolises the best linear separation using a SVM. The x-axis represents the score of the first fPC ζ1, the y-axis gives the score for the second fPC ζ2. All axes are scaled to the same size; shapes are therefore comparable. It should be emphasised that only the absolute slope value matters and not the sign (i.e. pointing upwards or downwards). (TIFF) [file pcbi.1011799.s010.tiff]

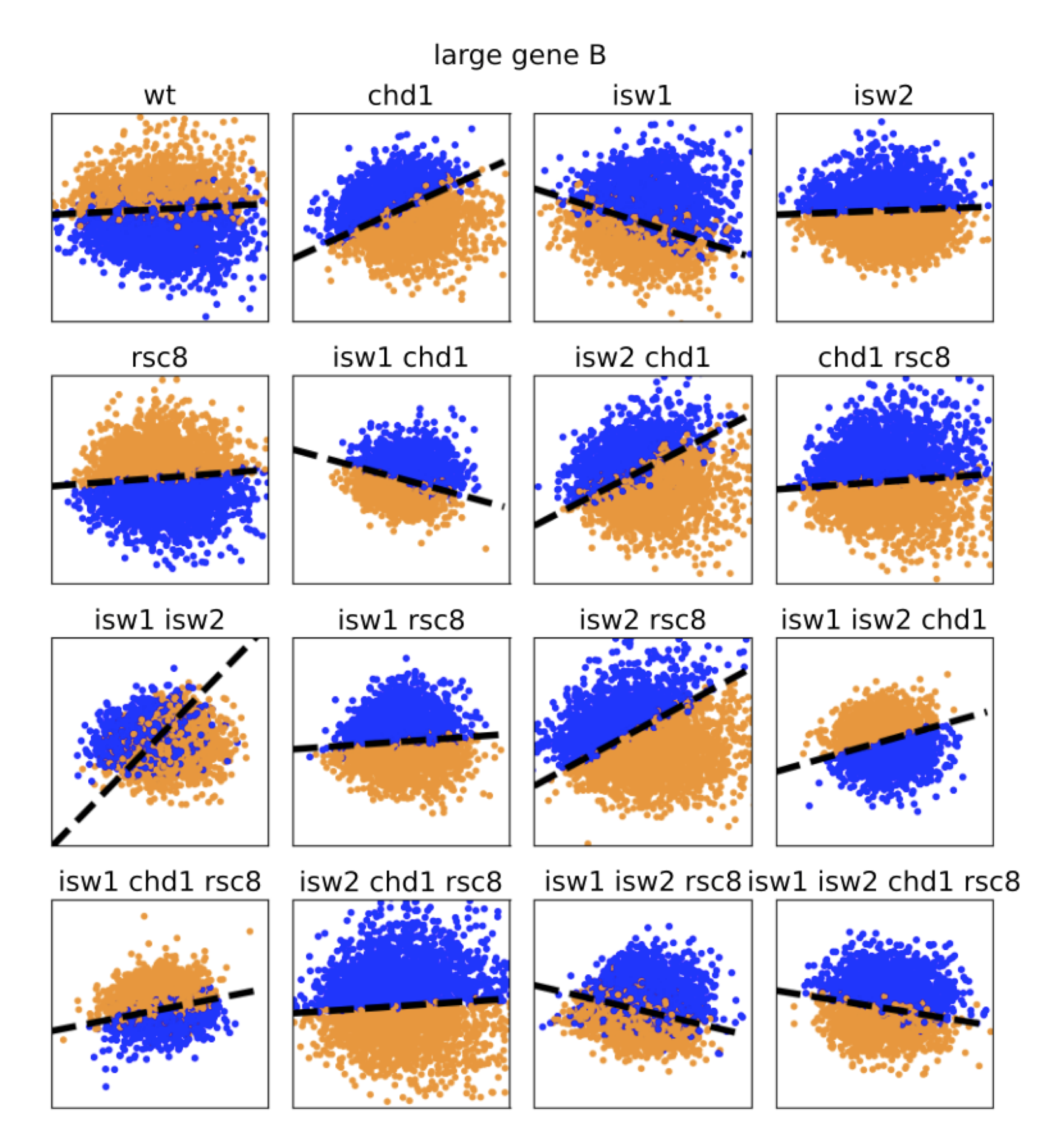

Supplement: S11 Fig — The figure shows the fPC scores ζ of all conditions coloured with respect to the Pearson clustering using only large genes (≥1000 bp). Blue and orange indicate each one group, the dashed line symbolises the best linear separation using a SVM. The x-axis represents the score of the first fPC ζ1, the y-axis gives the score for the second fPC ζ2. All axes are scaled to the same size; shapes are therefore comparable. It should be emphasised that only the absolute slope value matters and not the sign (i.e. pointing upwards or downwards). (TIFF) [file pcbi.1011799.s011.tiff]

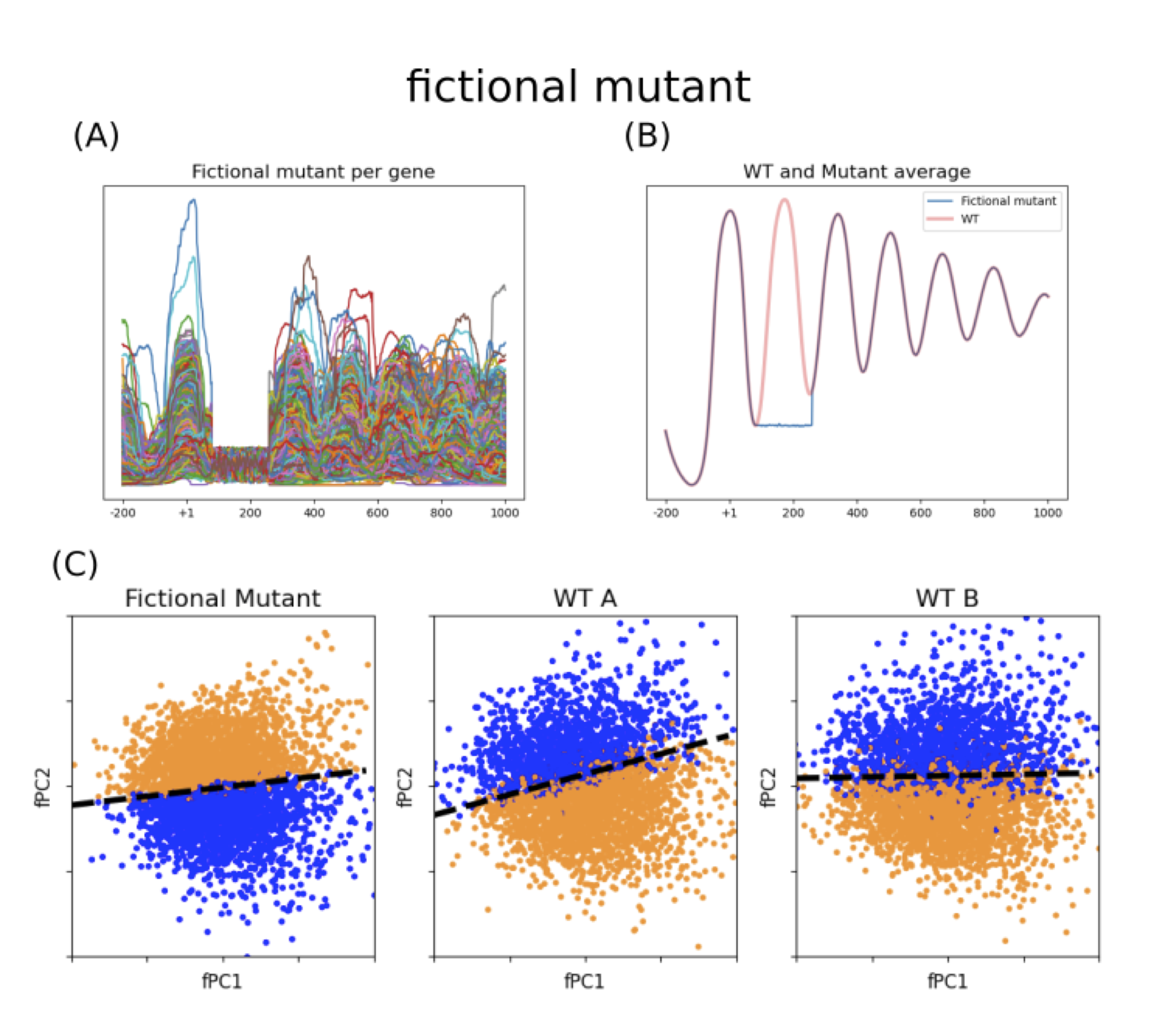

Supplement: S12 Fig — (A) We consider an arbitrary but fictional chromatin remodeler mutant that causes the depletion of the +2 nucleosome. All other nucleosomes remain unperturbed and keep their positioning. The MNase-seq profile at the +2 position consists only of random noise for all genes. (B) This has visibly a strong impact on the average distribution, as the +2 position is depleted in the arbitrary mutant (blue) in comparison with the WT (bold red line). (C) Since the descriptive variance decreased in our arbitrary mutant (i.e. variation that is not attributed to random noise), the fPC scores are slightly affected. Overall, however, the boundary slope remains fairly similar to the real WT A from which we constructed the arbitrary mutant. In fact, difference to WT A is smaller than the difference between the two biological replicates. In order to make sure we consider only mutants that affect the entire nucleosome array, we selected only strains for which the boundary slope notably changed with respect to the WT. (TIFF) [file pcbi.1011799.s012.tiff]

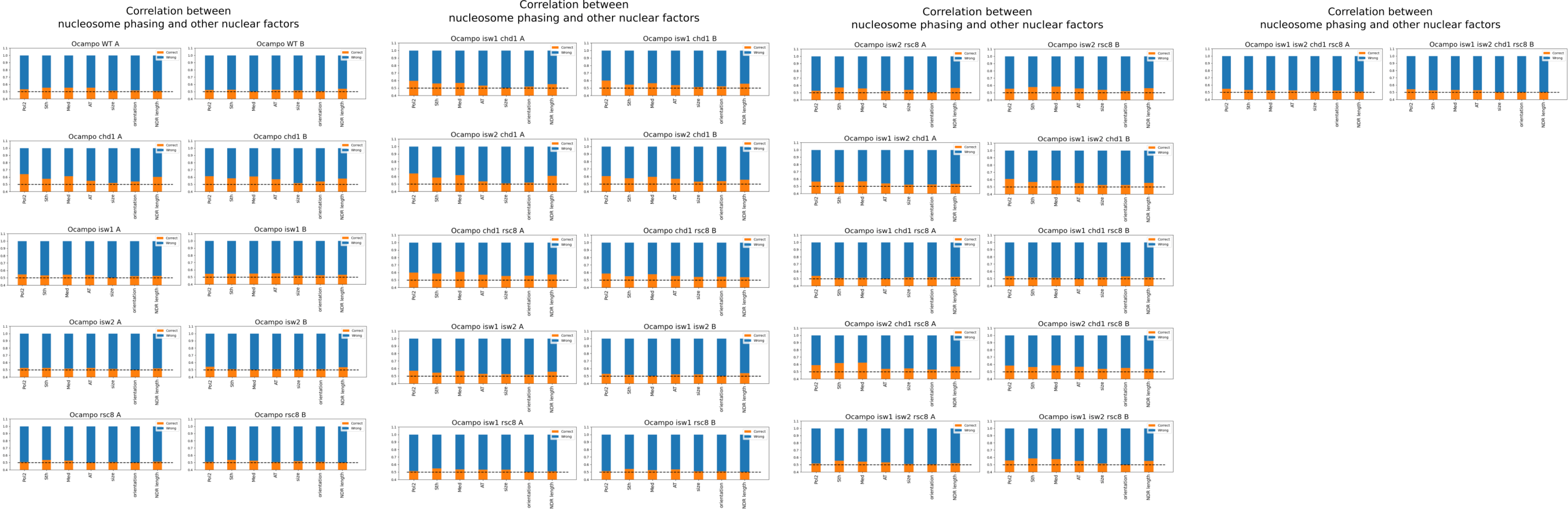

Supplement: S13 Fig — The orange bar shows the ratio of cases where the nuclear factor could predict clustering, blue gives the wrongly classified ratio. Random guessing would be correct in 50% of the cases, which is given by the dashed black line. Consequently, the orange bar must exceed the dashed line to suggest interdependence. (TIFF) [file pcbi.1011799.s013.tiff]

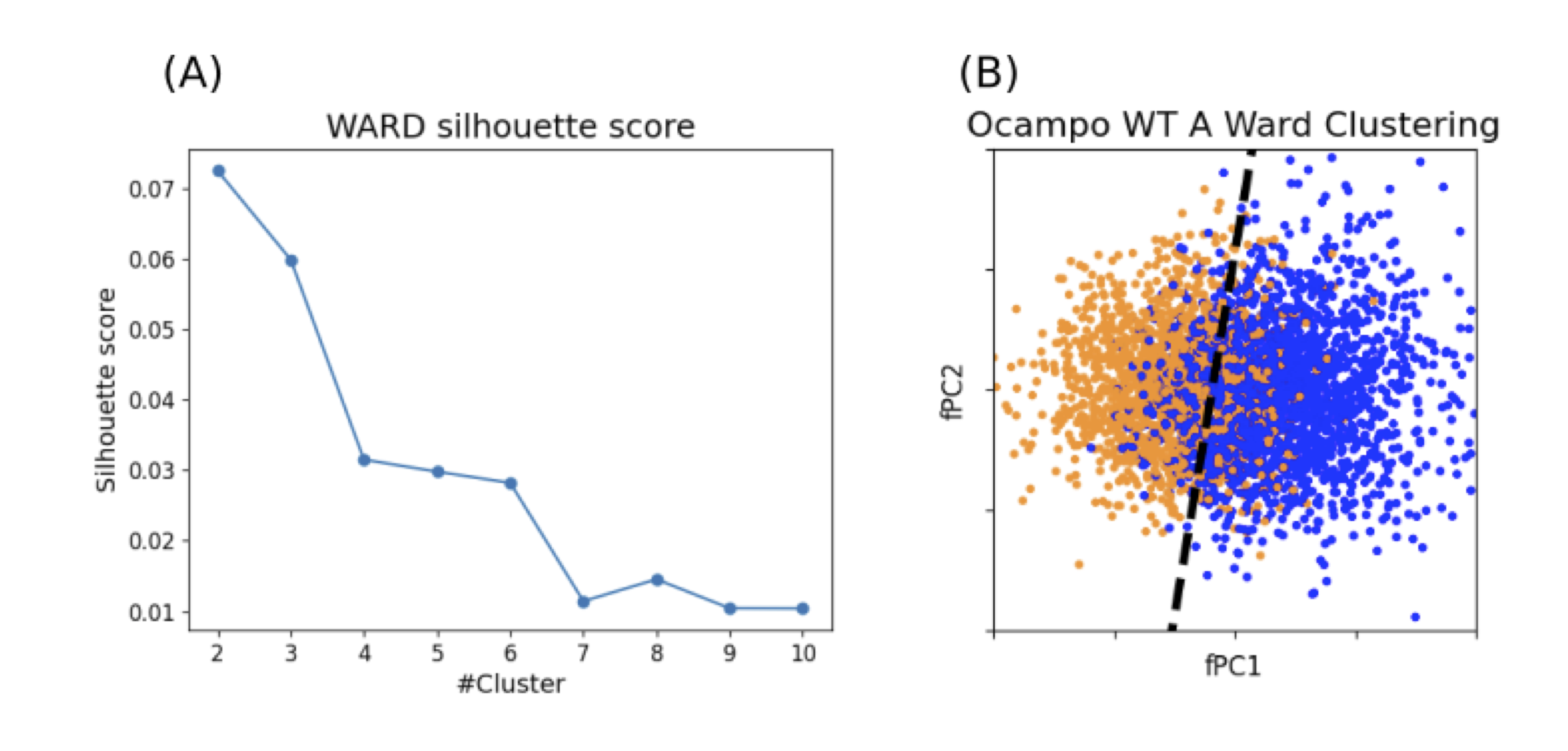

Supplement: S14 Fig — We repeated gene grouping (WT A, large genes) with WARD as a fundamentally different clustering method on the nucleosome profiles using an Euclidean distance metric. However, contrary to the Pearson correlation, the Euclidean distance does not have an upper bound, and clustering was more sensitive to outliers. We removed 29 genes who had an absolute fPC score larger than 20 for either of the two fPCs. (A) The silhouette criterion indicates once again that when dividing genes into clusters, it is best to separate them into two groups. (B) Despite the fact that the Euclidean distance measures a different property than the Pearson correlation and although WARD functions fundamentally differently, the gene groups tend to separate into two groups along the first two fPCs. The boundary is admittedly not as neat as for the k-mean clusters in WT A, but they are comparable to other mutants in the study. It is expected that the boundary itself changes, as the Euclidean metric captures different properties. We can conclude that the separation of nucleosome profiles into different gene groups along the major two fPCs is not merely an artifact of our methodology. (TIFF) [file pcbi.1011799.s014.tiff]

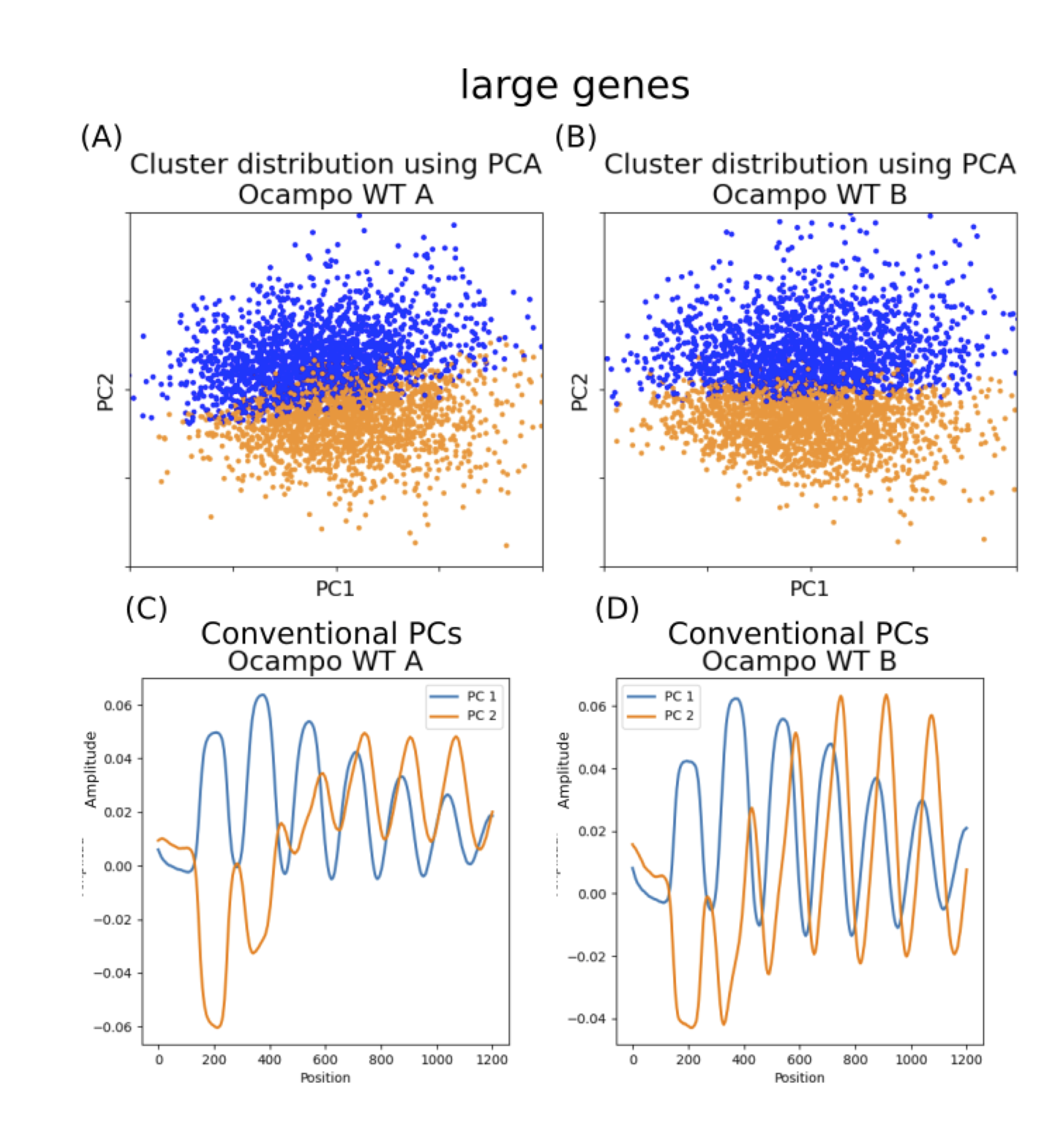

Supplement: S15 Fig — Indeed, conventional PCA can separate the clusters for all genes in WT conditions ((A) and (B) for replicate A and B) similarly to fPCA. The two clusters are given in blue and orange. However, the two determined PCs ((C) and (D) for replicate A and B) differ slightly with respect to the fPCA due to the independence assumption. Here, light blue and light orange indicate PC1 and PC2, respectively. (TIFF) [file pcbi.1011799.s015.tiff]

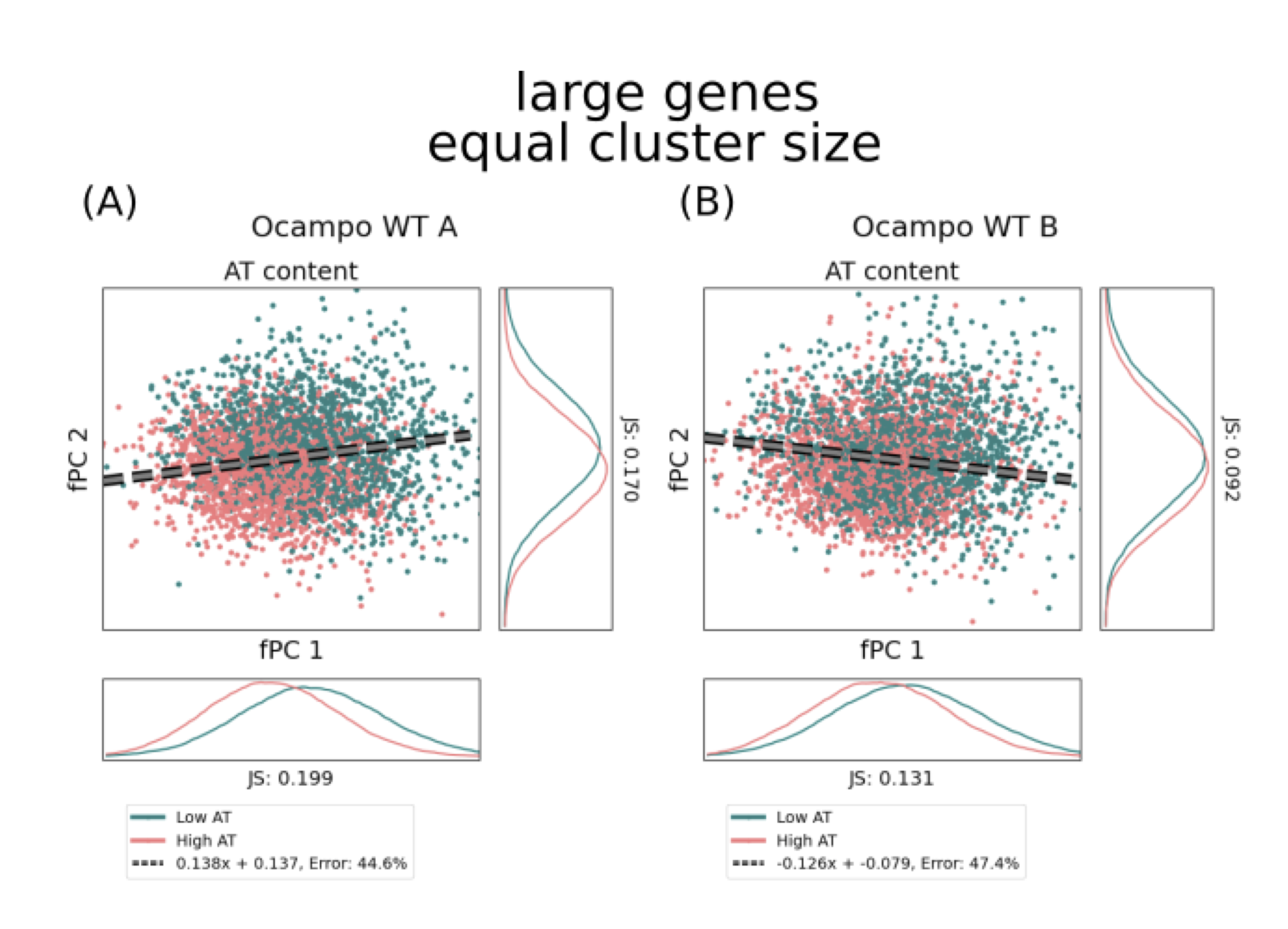

Supplement: S16 Fig — Whilst there is seemingly a slight correlation between Pearson coefficient clusters and AT-ratio in the A replicate, this is trend vanishes for the B replicate. In fact, both replicates might rather distribute AT-rich and AT-poor genes orthogonal to the dividing boundary. We plotted the original SVM boundary from the Pearson clusters with a dashed grey line to indicate that it was not determined using the AT content. (TIFF) [file pcbi.1011799.s016.tiff]
