# Supplementary material for: A genome-wide comprehensive analysis of nucleosome positioning in yeast
Source: PLoS Comput Biol. 2024 Jan 24;20(1):e1011799. doi: 10.1371/journal.pcbi.1011799 (PMC10843174; doi:10.1371/journal.pcbi.1011799)
Supplement: Attachment — Submitted filename: Response to reviewers Zeitler Nucleosome_JS.docx.pdf [file pcbi.1011799.s017.pdf]

Point-by-point response to reviewers' comments about the manuscript by Zeitler et al. A Genome-Wide Comprehensive Analysis of Nucleosome Positioning in Yeast (PCOMPBIOL-D-23-01024)

Introduction and general response to main concerns

We highly appreciated the constructive and valuable feedback of all three reviewers, which helped us to improve readability and accessibility of our results to a wider audience. In our work, we combined two computational approaches (i.e. the clustering of linear Pearson correlation indices and functional Principal Component Analysis (fPCA)) to evaluate collective nucleosome positioning in yeast. We admit that it was not always clearly stated (a) why we applied these approaches; (b) what information can be obtained by these two methods; and (c) which of both led us to specific conclusions. We incorporated the reviewers' suggestions and concerns with the main objective to clarify the aforementioned points. Here, all page numbers as well as figures and equations refer to the revised manuscript unless otherwise stated. Black font shows the reviewers' comments, and our replies are written in red. In the following, we summarise first the main changes in our manuscript, which is followed by a point-by-point response to each comment.

After reading the feedback, it became clear to us that the use of fPCA in our study was not sufficiently motivated and explained. FPCA itself was not used to cluster the MNase-seq profiles along genes. Rather, we applied fPCA to interpret the clusters that we obtained independently by performing *k*-mean clustering over Pearson correlations indices. The rationale is as follows. The Pearson value is a measurement to compare similarity of distributions. With respect to the wave-like structure of the MNase-seq data along genes, it indicates whether two genes are likely to exhibit a similar behaviour for coordination of the entire nucleosome array. By clustering these values, we divide all profiles into two groups which show the most distinct positioning (and therefore are likely to be formed by different dynamics). However, the Pearson correlation index itself represents only an average over the considered genomic region, and it is therefore difficult to interpret the clusters directly or to quantify why profiles have been allocated to either of both groups. In order to visually evaluate the two established sets of genes and how they can be separated, we apply a Dimensionality Reduction (DR) of the data itself (rather than on the Pearson correlations) and colour each profile with respect to its group. The clustering is completely independent of the fPCA. Therefore, the DR permits a direct comparison of the gene groups with the MNase-seq profiles, aiming to derive position specific properties for each cluster.

By using fPCA, we opted for a linear DR instead of a non-linear approach—often used for sequencing data—such as uniform manifold approximation and projection (UMAP) and t-distributed stochastic neighborhood embedding (t-SNE). We justify this by the fact that a linear reduction straightforwardly maintains the position-specific properties, which can be challenging for non-linear methods. We also preferred fPCA over conventional Principal Component Analysis (PCA) for the following reason. Whilst PCA fundamentally assumes independence between each position along the profiles, fPCA conjectures that the data was produced by a single stochastic process, and therefore assumes spatial dependence. Independence is problematic in our analysis because (a) we use a Pearson correlation index which analyses the entire profile as a whole and not each position individually; and (b) it

violates the main hypothesis of the well-established barrier model, in which nucleosomes are positioned with respect to a barrier and each other.

We particularly explained and highlighted these details in the Introduction (page 3), the first two Result subsections (page 3-7), and the Discussion (page 16-19). This also helped us to further motivate the choice of methods. In order to improve interpretability of the two clusters (or rather how they were established), we added a new type of figure for each strain in the third Result subsection (Nucleosome Phasing Changes In Remodeler Mutants, Figs 3-5). Here, we link the linear separating boundary that is detectable after the DR to location-specific properties along the nucleosome array, highlighting positions that were influential for clustering. We therefore complemented the main text in this section with an interpretation of these figures. We want to stress that despite updating minor observations and rephrasing several paragraphs in the Introduction, Results, and Discussion, all main findings remain unchanged. We updated our GitHub repository and added it to the manuscript (https://github.com/leoTiez/nucleosome-fpca). We also added several Jupyter Notebooks that produced the figures used in this response.

Reviewer #1:

1. Since the analysis mainly relies on Pearson coefficients, please include a brief description of their measurement and clustering in the main text and detailed information in the Method section. I recommend merging Fig 7 and Fig 1 for a comprehensive illustration of the process.

We added description and motivation in the beginning of the Results section (page 3 and 4, lines 128-137), and a more detailed mathematical explanation in Methods (page 20, lines 624-629). We added previous Figs 7(B, C, D) with Fig 1 and removed previous Fig 7(A) from the manuscript.

2. Clarify the rationale for clustering Pearson coefficients and how it aligns with the interpretation of the two Pearson clusters.

As mentioned in our general response, we added a detailed motivation about the Pearson correlation index, the clustering, and their meaning in the Results (page 4, lines 138-163) and Discussion (page 16, lines 473-487)

3. What is the justification for using 20 B-splines, and how does it compare to conventional smoothing techniques?

We aimed to find a functional representation of the data that finds a good balance between flexibility (in order to fit well each nucleosome profile) and constraint (to avoid overfitting and including too much signal noise). When comparing different numbers of B-Splines (i.e. 5, 10, 20, 50, and 100), we can show that the separation between the two clusters does not change anymore when using the first two fPCs from 20 B-Splines onwards (see Fig 1 for Reviewers). In order to measure the trade-off between flexibility and constraint, we divided the Mean Squared Error (MSE) by the degrees of freedom (i.e. 1200 base pairs (bp) length of the considered profile minus the number of used B-Splines). This is comparable to a conventional ANOVA analysis. Indeed 20 and 50 B-Splines exhibited a lower value (0.10836 and 0.10659, respectively) than 5, 10, and 100 B-Splines (0.16331, 0.15649, and 0.11143,

respectively). As we deemed 20 and 50 B-Splines sensibly similar and because 20 B-Splines do not affect the linear separability of the clusters (Fig 1 for Reviewers), we applied Occam's razor and opted for the lower number of B-Splines. We also compared 20 B-Splines with 20 Fourrier functions, both of which produced very similar results (0.10838 B-Splines vs. 0.10707 for Fourrier functions). We therefore presume that 20 B-Splines is a sensible choice.

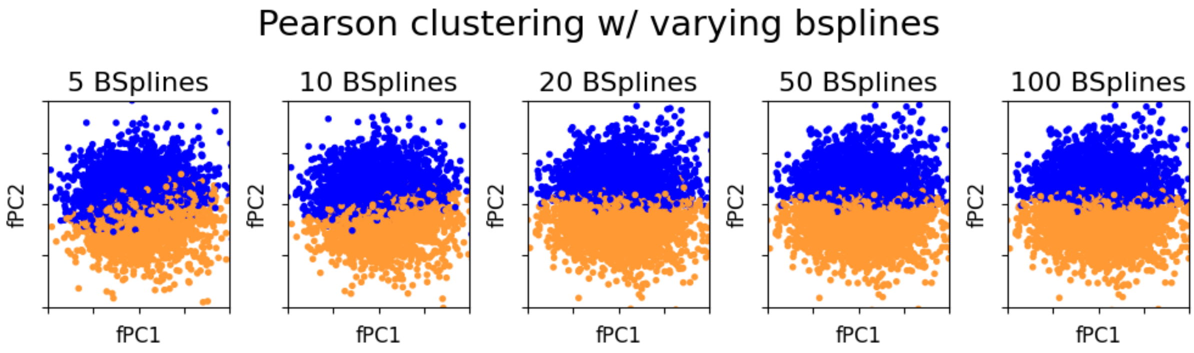

Fig 1 for Reviewers. The 5 plots show the Pearson cluster distribution with respect to the gene-specific factors of fPC1 and fPC2 for 5, 10, 20, 50, and 100 B-Splines in WT using all genes. Although the Pearson clusters can already be roughly discriminated using only 5 and 10 B-Splines, the separation is improved until using 20 B-Splines. Considering more B-Splines does not improve the separability. Orange and blue indicate the two Pearson clusters. The axes represent the gene-specific factors for the fPCs. All axes are equally scaled.

4. Could the framework's effectiveness be compromised if the MNase-seq signal doesn't align with a wave-like distribution (lines 129-132)?

By itself, the Pearson correlation measurement as well as the fPCA are independent of the profile shape. Therefore, they can be applied to any distributional data, including other NGS signals. However, we leverage the wave-like property in order to interpret the separability of the two clusters with respect to the determined fPCs. Due to the repeating peaks, we can presume that the Pearson cross-correlation measures either differences in exact positioning, a trend over the profile (i.e. increasing or decreasing presence into the gene body), or a combination of both (Fig 1(D)). This consideration is important in order to make conclusions about the collective behaviour of the entire nucleosome array as one entity. Initially, it also simplified identifying important positions that influence the clustering (i.e. associating properties of the fPCs to either trend or shift). In the revised version of the manuscript, we include additional figures to provide more insights about how the clusters can be separated by highlighting influential positions along the nucleosome arrangement (i.e. Figs 3(C, F), 4(C, F, I), and 5(C)).

5. Explain the choice of the first two functional Principal Components (fPCs) for nucleosome profile analysis (line 138). What criteria were used, and will the third fPC provide additional insights?

Similar to conventional PCA, the orthonormal eigenfunctions are established one after each other, starting by determining the fPC that explains most variance in the data, then the second until all *m* independent eigenfunctions were calculated, with *m* being the number of

basis functions, as explained in the Method subsection Functional Principal Component Analysis by Eqs 7 and 8 (Eqs 4 and 5 in the previous version of the manuscript). Therefore, the first two fPCs are the orthonormal eigenfunctions that explain most variance in the data. Undoubtedly, we would include more information by adding the third fPC, as the profiles could be better described. However, the third fPC does not provide additional insights for interpreting the clusters, since they can be already visually separated using only the first two fPCs (see Fig 2 for Reviewers). As our declared goal is to describe the differences between the two Pearson clusters and not to find the best data representation, adding more fPCs does not improve our analysis. We added more details to the manuscript in Results (page 6 lines 210-213 and page 7 lines 274-276) and Discussion (page 17, lines 517-519).

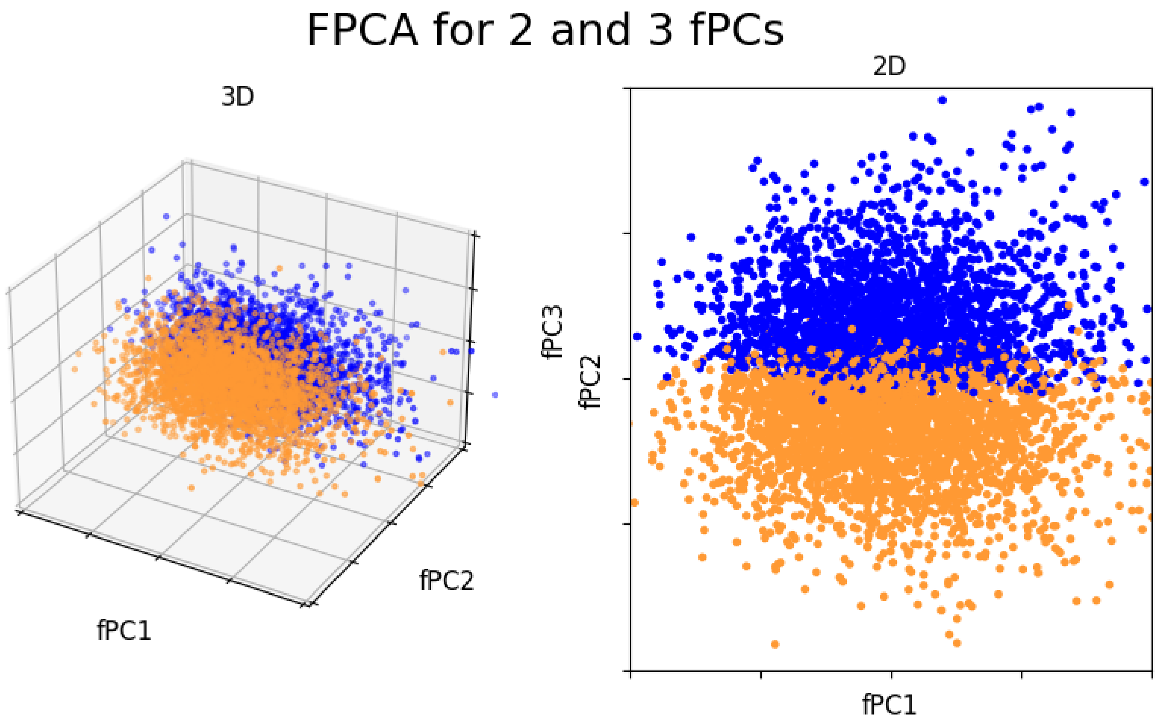

Fig 2 for Reviewers. Although considering more fPCs could possibly improve the approximation of nucleosome profiles in WT conditions over all genes, they do not provide more information to explain the two Pearson clusters and their separability. The two clusters are coloured in orange and blue, the axes represent the gene-specific factors for the fPCs. All axes are equally scaled.

6. In result2, disclose the ratio between small and large genes. Does the majority of large genes affect the similarity with all-gene fPCs?

We added the ratios to the main text (page 6, lines 218-219). Most of the genes are larger than 1000 bp (73.3%). As fPCs are established over the entire data set (including genes that are smaller than 1000 bp and whose profile therefore do not follow the same wave-like shape), both gene-size groups influence the result. Due to the fact that most of them are coding regions bigger than 1000 bp, the variance over all profiles is also dominated by large genes. This is an important aspect, as we show that the linear separation only changes slightly. Consequently, Pearson clusters established with respect to all genes or only large genes measure similar gene-specific nucleosome phasing dynamics.

7. In line 155, elaborate on the size-dependent phasing of nucleosome positions with rsc8-depleted strain. Are the results consistent with the mutation data in the authors' recent publication?

We interpret the fact that the two Pearson clusters over all genes can be linearly separated using either fPCs over small genes or all genes as follows. We conjecture that the Pearson correlation indices represent similarity in phasing dynamics over an entire nucleosome array of a 1000 bp after the +1 position (considering the arrangement as a single entity). Consequently, the separability indicates that genes smaller than 1000 bp conserve the same dynamics. This suggests that nucleosomes outside the considered coding region are positioned with respect to nucleosomes inside the gene. From this perspective, it is not only the previously reported size of the Nucleosome Depleted Region (NDR) that shrinks upon depleting *rsc8* (as this could nonetheless conserve independent phasing dynamics within gene boundaries). Rather, the remarkable regularity of peaks in the second fPC of small genes suggest that a discrimination between an arrangement inside from outside is completely lost. Indeed, our own recently published data with WT and a conditional thermosensitive (ts) *rsc8* mutant strain *(André et al., 2023)* indicate the same conclusion (Fig 3 for Reviewers).

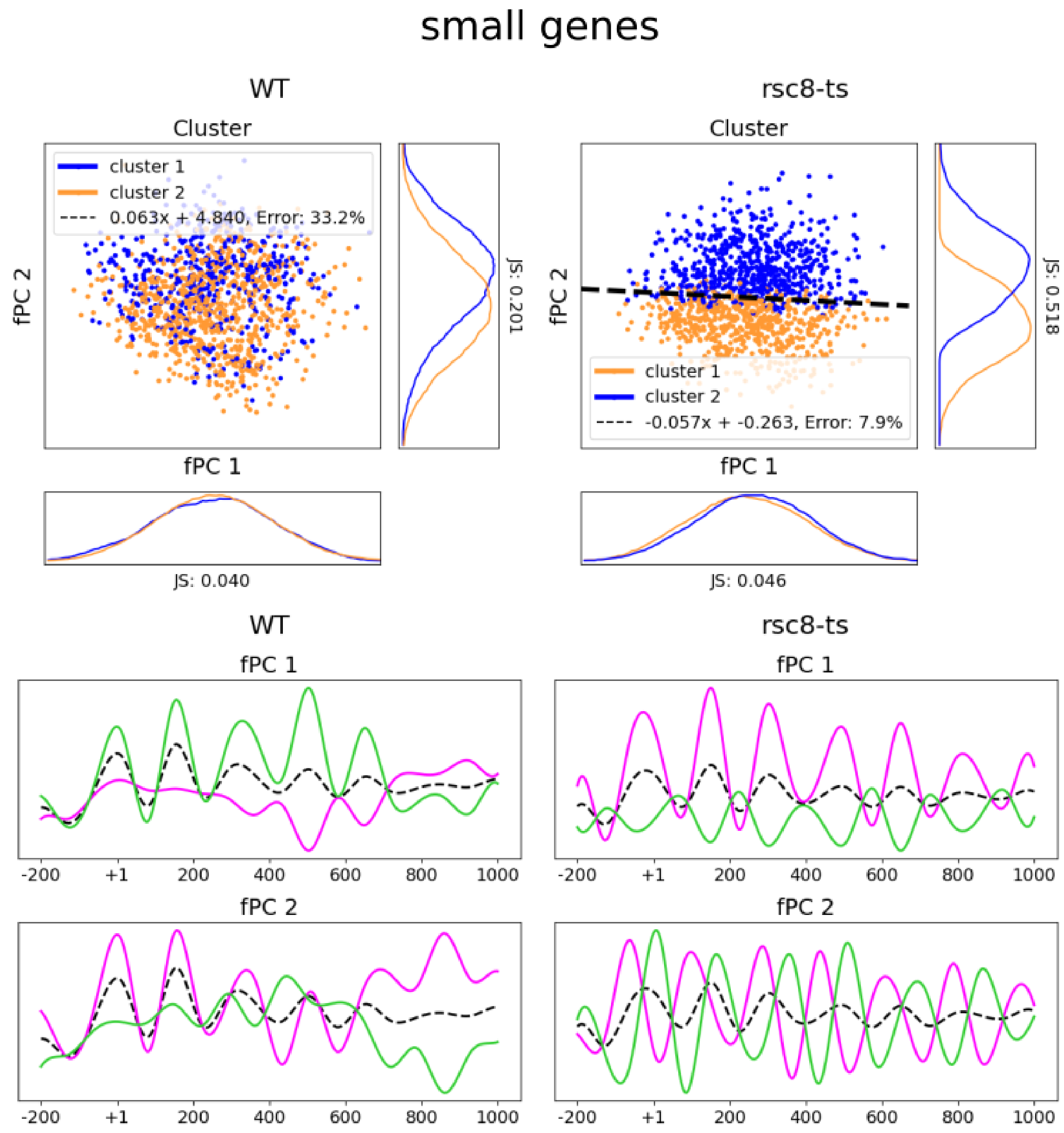

Fig 3 for Reviewers. When considering the all-gene clusters over exclusively small genes, using the data produced by the laboratory and published by André et al (2023) shows similar results as the analysis for the data by Ocampo et al. (2019). This is even true despite the fact that the *rsc8* strain in our recent paper (André et al 2023) is thermosensitive (ts), which is not the case for the data by Ocampo where *rsc8*-depleted strain was used. Left shows the results for WT cells, right is *rsc8-ts*. Top row displays the Pearson cluster distribution with respect to the fPCs (orange and blue representing the clusters), and the bottom row shows the transformation of the mean by the two fPCs (black dashed mean, positive contribution magenta, negative contribution green). The plots were created as for the presented manuscript.

8. Consider shifting SFig A6 to the main figure, possibly merging it with Fig 2 to clarify Chd1 and Rsc8 roles.

We updated Fig 2 by including results for *rsc8 chd1* and added more explanations to the caption to set *rsc8*-depleted and *rsc8*-depleted *chd1*Δ into context. In particular:

> *[...] (B) The fPCs of the rsc8-depleted strain maintain the linear separability, despite the fact that the groups were established for all genes. As we interpret the Pearson clusters as similarity in positioning between genes of 1000 bp mediated by chromatin remodelers, it possibly suggests that positioning outside coding regions influences nucleosomes inside and vice versa. (C) Whilst most mutants that were rsc8 depleted could discriminate between the all-gene clusters using small-gene fPCs, this separability is lost again in rsc8-depleted chd1Δ, revealing partly antagonistic roles to maintain gene-specific phasing for Rsc8 and Chd1. [...]*
> *(E) Nucleosome positioning in rsc8-depleted conditions is clearly visible along the entire considered region, despite the included genes being smaller. This suggests that gene-specific nucleosome arrangement cannot be maintained. It is of note that the phasing also changes for the +1 nucleosome, and the NDR can be seemingly not conserved. (F) rsc8-depleted chd1Δ on the other hand loses the wave-like form of its second fPC after the +2 nucleosome, indicating the presence of gene-specific nucleosome profiles as in WT conditions. [...]*

9. In line 204, detail how gene size-dependent bias removal was confirmed and discuss how other potential biases were addressed in key factor comparisons.

The Results subsection FPCA Reveals Size-Dependent Rsc8-Mediated Phasing of Nucleosome Positions showed that nucleosome phasing within genes is decoupled from phasing outside. As fPCA establishes the orthogonal eigenfunctions considering the entire profile (200 bp before the +1 and 1000 bp after) and is performed over all genes, the positioning outside coding regions smaller than 1000 bp distorts the result. As shown in SFig A5 (SFig A4 in the previous version), the two fPCs represent to a large degree the gene size, and making a link to collective nucleosome phasing of the entire array is not possible. Indeed, the fPC2 considering all genes (Fig 1(F)) is slightly different from the second fPC considering only large genes (Fig 3(B)), particularly with respect to +3 nucleosome. The fact that the +3 position is better defined for large genes visualises the impact of small genes, whose fPC2 loses the wave-like shape after the +2 nucleosome (Fig 2(D)). Using our new figures that display the position-specific effect of the separating boundary along the entire profile, we can show that the fPCs change sufficiently much to change the interpretation of cluster-influencing locations (compare Fig 4 for Reviewers (A) and (B)). Nonetheless, the two Pearson groups are dominated by large genes, and therefore do not affect the evaluation of the separating boundary (Fig 4 for Reviewers (C)). In order to avoid a gene size-specific bias of our interpretation of the two clusters, we removed small genes from the fPCA. To be sure that they do not influence the *k*-mean clusters either, we also repeated the grouping (lines 267-269), although results for WT indicate that they are similar (Fig 4 for Reviewers). No other bias was considered at this stage. It should also be noted that at this point, we did not restrict the size of the clusters themselves (i.e. how many profiles are in each cluster). This was only imposed when comparing the gene groups (that represent different phasing dynamics), to other genomic properties (Section Pol II Presence Correlates With Nucleosome Organisation in chd1Δ Mutants in Results).

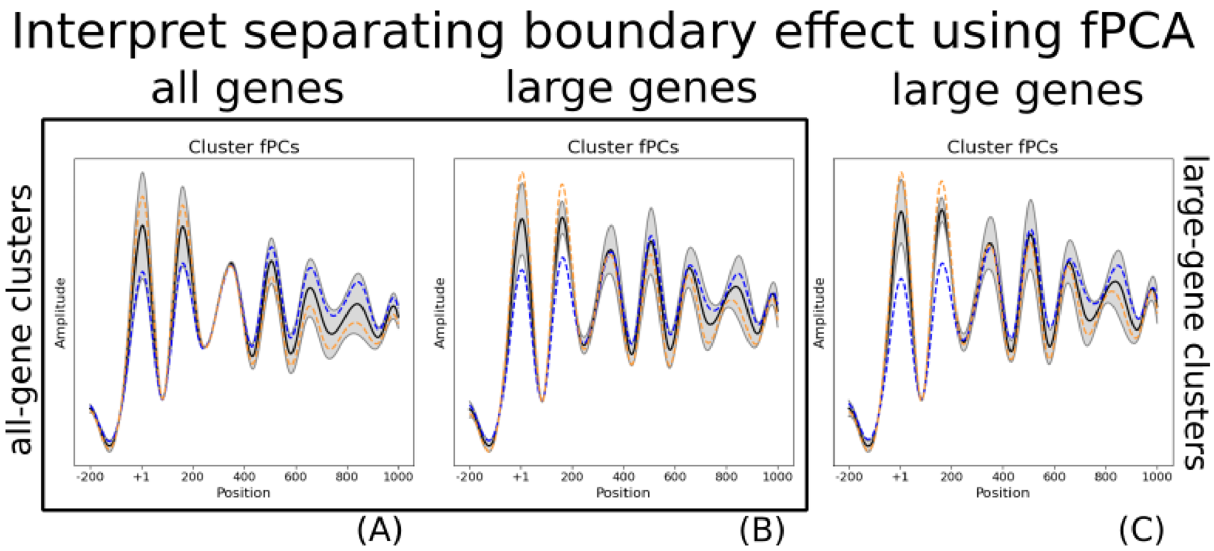

Fig 4 for Reviewers. When considering the effect of the all-gene clustering with respect to all genes (A) or large genes (B), we obtain different results. Whilst for clustering all genes the +3 nucleosome is not important, it plays an important role when considering only large genes (compare grey areas). When the clustering is also performed using only large genes, the location-specific impact of the separating boundary remains unaffected (C). Blue and orange indicate the median profile of cluster 1 and 2, respectively. Black is the mean. The grey areas show the location-specific impact of the separating boundary between cluster 1 and 2. It can be interpreted as highlighting important areas the larger the margin is.

10. Explain the removal of isw1Drsc8D from the analysis in lines 207-208. What makes them exceptions?

As mentioned in the main text, we evaluate the significance of the two established Pearson clusters by comparing their similarity with 500 random group pairs. It is expected that the distance between the Pearson clusters is significantly larger than of the 500 random groups. Distance was measured using the Jensen-Shannon (JS) divergence. We approximated the distribution over the JS distance of 500 random group pairs using a gamma distribution. If the JS distance between the Pearson clusters was outside the 95% Prediction Interval (PI), the clustering was seen to be significant (explained in Results (page 4 lines 144-147 and page 7 lines 270-271, page 3 lines 114-117 and page 7 lines 207-207 in the previous version), Discussion (page 16 lines 480-487, page 13 lines 347-353 in the previous version), and Methods (page 20 lines 637-652, page 16 lines 463-471 in the previous version)). *isw1Δrsc8* was removed because the established clusters were not outside the 95% PI of the estimated gamma distribution (SFig A.8 (part 2)), indicating that random group pairs could possibly create Pearson clusters that are sufficiently dissimilar. This suggests we cannot sensibly distinguish between different phasing dynamics. We added more details to the Methods section (page 20, lines 637-652).

11. In line 313, clarify the differences between replicate A and B, as they may be due to the nonlinear effect of double mutants, not just "experimental variability."

Replicate A and B are two replicates of the same experiment and of the same strain. Therefore, any nonlinear effect evoked by the mutation should be identical in both. Replicates are commonly used to quantify the variability that can be observed.

12. Regarding lines 234-236, clarify the results leading to the conclusion about notable alterations in coordinated phasing. Please elaborate on why the focus is on Chd1 and its effect after the +1 nucleosomes.

In the preceding lines in the previous version, we established that phasing within the gene body is altered with respect to the WT, whereas the +1 nucleosome remains well positioned. In lines 234-236 of the previous version, we wanted to make a link between our conclusions and the known effects of Chd1 on nucleosome phasing from the published literature that we presented in the Introduction. In the revised version, we included additional details and insights to make our point clearer (page 9, lines 332-333):

> *As Chd1 is responsible for nucleosome spacing along genes and is particularly involved in maintaining chromatin integrity during Pol II elongation, it is intuitive that the chd1 -deletion influences phasing within the gene body. This outcome shows the clear effect of chromatin maintenance by Chd1 after the +2 nucleosome, whilst leaving the +1 position well preserved.*

13. In the last result, consider presenting a correlation or causal relationship between remodeler deletions and genomic properties, perhaps using Bayesian network or structural equation modeling, if possible.

In the Result section Pol II Presence Correlates With Nucleosome Organisation in chd1Δ Mutants (page 13-16), we evaluate a possible direct causal relationship between genomic properties and the established Pearson clusters that represent collective nucleosome positioning. Our top-down analysis is purely based on the data since we wanted to avoid including additional assumptions about indirect or hidden influences, as they are commonly represented in structural equation modelling. This would be out of scope for this project. Furthermore, Bayesian networks is a graphical model that indicates conditional dependencies between model variables, which were not directly available to us. Rather, we understand our study as a purely data-driven analysis. Nonetheless, a structural modelling approach is currently being developed by the laboratory, which might be part of a future publication.

Minor Concerns
1. Verify the reference in line 181 to "SFigs A.2(G, H)," as it seems to be missing.

Thanks for pointing that out. We corrected it in the revised version.

Reviewer #2: The question of how different remodelers move nucleosomes is of fundamental importance and has not been completely understood. Here the authors have performed a reanalysis of the experimental dataset of Ocampo et al using a new computational method of functional PCA. I think, this is an interesting method, which should work fine theoretically. I think it is important to prove that this method is reproducing the

results obtained with other methods like classical PCA or other clustering which has been applied to MNase-seq analysis previously. On the other hand, if the results are different, then it would be good to explain what's different, why, and what's the biological meaning of the clusters that are uniquely identified by fPCA and cannot be found by other clustering methods. More detailed suggestions are listed below in the order of the manuscript flow.

This is an important and good point that is raised here by Reviewer #2, as we realised that we did not sufficiently highlight the difference to other DR methods. As mentioned in our general response above, we substantially revised our manuscript to motivate the use of fPCA and why we prefer it over other DR methods, such as conventional PCA. However, we want to highlight again that fPCA was not used for establishing the clusters themselves. Rather, it was used to interpret the gene groups and how they were found, which was performed independently using *k*-mean clustering on the pairwise Pearson correlation indices. We added a comparison with conventional PCA to the Supplementary Data (Fig A12) which is discussed on page 17, lines 488-506.

Lines 60-62: "It has been suggested that high gene expression correlates with low nucleosome regularity, which is characterised by weak phasing and extreme spacing (both short and long) [18, 28]." I think this statement contradicts the conclusions of Ref 31 which shows stronger phasing at highly expressed genes. Refs 18 and 28 cited here probably also do not support this statement in this form.

We thank Reviewer #2 for pointing out the results in Ref 31 (*Baldi et al, 2018*). First, we want to emphasise that we define nucleosome *positioning* and *phasing* to be the positions of the MNase-seq signal peaks over an entire single nucleosome array. We added the definition to page 3 (lines 89-90) to avoid any ambiguity.

We noticed that some publications use different definitions. We rephrased our statement such that the definition of the cited publication can be applied (lines 62-67, below citation [30] changed to [31] to use the same reference as used by Reviewer #2):

> *It has been suggested that high gene expression correlates with low nucleosome regularity [28] as well as extreme spacing (both short and long) [18]. There are contradicting results about the correlation between transcription and nucleosome phasing. Whilst [18], [29], and [31] report that transcription increases random positioning and weakened phasing, [28] show that nucleosome phasing of highly expressed genes is increased.*

Using this rewording, we are convinced that the linked references do support our claims. Although they focus particularly on regularity and spacing, they also indicate that gene expression weakens phasing. Below we cite directly from the papers Ref 18 (*Ocampo et al., 2016*) and Ref 28 (*Koerber et al., 2009*).

Ref 18 (*Ocampo et al., 2016*):

> *Abstract page 1: Heavily transcribed genes show weak phasing and extreme spacing, either very short or very long [...]*

Page 2: We confirm that transcription is associated with short spacing [Citation deleted], but we also find that some heavily transcribed genes have extremely long spacing.

Page 4: This analysis confirms that heavily transcribed genes have extreme spacing in wild-type cells and shows that the same is true for all of the mutants (Figure 3B). The large majority of genes in the normal range of spacing are much less transcribed. Thus, rather surprisingly, heavy transcription is associated with very long spacing as well as very short spacing.

Page 5: In wild-type cells, active genes have much weaker phasing and shorter spacing than the average [...]

Page 9: Heavily transcribed genes have a wider NDR, decreased nucleosome occupancy and disrupted phasing on gene bodies [Citation deleted].

Ref 28 (*Koerber et al., 2009*)

Abstract page 1: We find that a transcribing RNA polymerase creates a delocalized state of resident nucleosomes.

Page 8-10: The genomic locations of Pol II-crosslinked nucleosomes were determined by sequencing 5,097,371 of these nucleosomes (Figure 6D) and were verified by hybridization to high-density tiling arrays. Size selection after MNase digestion (Figure 1B) ensured that intact nucleosomes were being examined. The genes associated with the top 150 peaks were analyzed further (Figure S5A and listed in Tables S1 and S2). The positions of the Pol II-crosslinked nucleosomes lacked phasing (Figures 6D, 6E, and S5B), so making consensus calls of their positions was not informative. Individual nucleosomal tags were not enriched at canonical locations, as evidenced by a lack of well-defined peaks and valleys of tags around the TSS (Figures 6F and S5C). Genes that contained relatively high levels of Pol II-crosslinked nucleosomes were generally highly transcribed (red bars in Figure 6E) and depleted of nucleosomes. We interpret these findings to suggest that, during transcription, Pol II collides with nucleosomes (detected as Pol II-crosslinked nucleosomes), and this results in their random repositioning and ultimately their eviction or partial dismantling to allow passage of Pol II (hence, nucleosome depletion).

We found another study that supports our claim that positioning is disrupted when transcription is induced (*Cole et al., 2011*), which we added to our revised manuscript.

Abstract page 1: Induction results in a major disruption of nucleosome positioning, sometimes with altered spacing and a dramatic loss of occupancy over the entire gene, often extending into a neighbouring gene.

Page 6: Thus, ARG1 induction was associated with loss of more than half of its canonical nucleosomes; those remaining were no longer organized into clusters with dominant positions.

Indeed, as claimed by Reviewer #2, Ref 31 (*Baldi et al, 2018*) comes to slightly different conclusions. Although our statement about decreased phasing of highly expressed genes is indeed not necessarily supported by this study, the claims about low regularity is replicated, as indicated by the following sentence taken from the abstract:

We also found that nucleosome arrays downstream of silent promoters are considerably more regular than those downstream of highly expressed ones, despite more extensive nucleosome phasing of the latter.

We want to point out that this result was obtained by Array-seq in *Drosophila melanogaster* instead of MNase-seq in yeast.

Due to these slightly contradicting conclusions, we sorted the profiles over large genes with respect to the Pol II occupancy over the considered 1200 bp (using the same Pol II ChIP-seq data as in our study). We can report that the data by *Ocampo et al. (2016, 2019)* rather support reduced phasing of highly transcribed genes (Fig 5 for Reviewers).

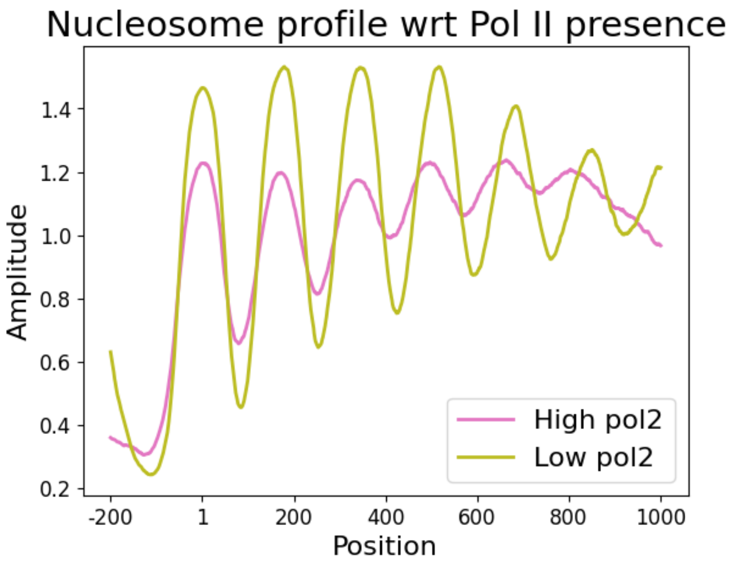

Fig 5 for Reviewers: When averaging the nucleosome profiles of large genes with the highest and lowest 15% Pol II presence over the considered 1200 bp, the data indicates weakened phasing in genes with high Pol II presence. Pink displays high Pol II occupancy, light green shows the profile for genes with low Pol II occupancy.

Lines 78-81: “To our knowledge, a single mathematical framework assessing gene-wide nucleosome phasing has not been proposed, and a direct comparison of the effects in different remodeler-deficient strains is difficult”. I am not sure what is expected as a "single mathematical framework", but a number of publications, including e.g. Ref 31 cited here, developed different frameworks for this purpose.

With “a single mathematical framework” we mean that the same set of mathematical methods can be applied in the same way independently to all strains in order to interpret nucleosome positioning dynamics along the entire arrangement with position-specific properties. Ref 31 (*Baldi et al., 2018*) developed an experimental method (not a mathematical framework), and their analysis does not include mutant strains. We changed the phrasing to make our point clearer (page 3, lines 83-87)

> *In order to provide comparability of nucleosome positioning changes between various mutants, we aimed to use a single mathematical framework that can be applied to all strains. To our knowledge, a unifying approach assessing location-specific phasing properties along the entire nucleosome array over varying conditions has not been proposed, and a direct comparison of the effects in different remodeler-deficient strains is difficult.*

Lines 83-85: “By doing so, we address two points that have previously largely fallen short. Firstly, we evaluate long-range influences of positioning within transcribed regions that exceed neighbouring nucleosomes”. Autocorrelation-based approaches also consider distances between nucleosomes beyond next neighbours, so this statement needs to be refined to explain the difference of the current approach a bit better.

We agree that this statement needs more detailing. We removed the reference to long-range influences, and we substantially rephrased our motivation on page 3 in the revised manuscript (lines 105-112):

> *The established Pearson clusters can be visually separated by considering only two fPCs, which are therefore sufficient to interpret the gene groups. Using our analysis, we can repeatedly investigate histone complex distributions of different chromatin remodeler-mutant strains using the same framework and interpret major differences along the entire nucleosome arrangement. By relating Pearson correlation with spatial properties along the profiles, our approach refines and complements other studies that focused either on a few individual nucleosomes close to the NDR or Transcription Starting Site (TSS); or which assessed only the average correlation of the entire array (e.g. via autocorrelation).*

Lines 129-130: “..the link between linear correlation and coordinated 129 nucleosome phasing.” Linear correlation of what?

Here, we refer to the pairwise linear correlation of MNase-seq profiles along 1000 bp after and 200 bp before the +1 position of coding regions that were measured and with respect to which the clusters were established. The context of this phrase was to provide additional insights into how to interpret these two clusters, and what the implications are if the signal does not follow a wave-like structure. As this phrasing seemingly caused confusion, we removed the sentence in this paragraph and added a short explanation where it was needed for the triple and quadruple mutants on page 11 (lines 376-379).

Figure 1: I suggest to add the percentage of explained variance to each principal component on the axes. Also, please rewrite the legend to explain what exactly is schematically drawn or plotted in each panel.

We changed Figure 1 as suggested by Reviewer 1, and added more details to the legend. We added the explained variance ratio to the main text on page 6 (line 211). However, we refrain from adding these values to the plot itself, as they are irrelevant for our actual analysis. Instead, we explain the use of only two fPCs on page 6 (lines 210-213):

> *Despite the fact that the ratio of explained variance is not high (21.4% and 11.5% for fPC1 and fPC2, respectively), they are completely sufficient to distinguish between the Pearson correlation groups and permit an interpretation of the linear separating boundary between the clusters.*

Line 144: “Astonishingly…” Unclear why this word is used.

We wanted to emphasise the fact that the gene clusters—which were independently established by *k*-mean clustering over all pairwise Pearson correlation indices—can be neatly separated by only two fPC—which allow a new data representation that explains the largest variance in the data and have nothing to do with the clusters themselves. We removed this word in the revised version.

Figure 2: Panels A-C here seem to report the main finding of the disappearance/reappearance of the cluster separation. Please try to reproduce the effect of disappearance/reappearance of the cluster separation using some other method (e.g. perform k-means clustering with one of standard software packages that exist for classical MNase-seq analysis), or explain why it can not be reproduced using another method. If it can not be reproduced with other methods it is really important to explain what these clusters represent, both mathematically and biologically. Are they characterised by different averaged nucleosome profiles? Then it would be good to show such different averaged profiles in the manuscript.

At no point, the fPCA has been used for the clustering itself. As explained in the first subsection of the Results (Nucleosome Profiles Can Be Well Distinguished Based On Their Coordinated Positioning in WT), we established the two gene groups exclusively using *k*-mean clustering on the pairwise Pearson correlation coefficients (therefore using the recommended approach). The clustering was performed in MATLAB, therefore using well established and tested algorithms. We assume that we made our motivation for combining clustering over Pearson indices with fPCA not sufficiently clear in the previous version. As explained in our general response above, we substantially revised the manuscript to make motivation, interpretation, and consequences of our approach clearer. We hope that the new version removes the concern raised by Reviewer #2.

Line 207: The authors have removed some data as outliers. It would be good to list the total number of datasets before/after the removal of this outlier dataset.

Each strain was analysed individually. The slope between the clusters for each cell line and replicate as well as their mean slope and significance value as defined in Eq 9 (Eq 6 in the

previous version) were given in Table 1. Eq 9 considers both replicates for isw1Δrsc8, and its outcome was lower than 1, indicating that the slope over the Support Vector Machine (SVM) boundary was not sufficiently different from WT (also reported in Table 1). As the two Pearson clusters were not sufficiently significant, we did not further investigate the effects of this strain.

Figures 3, 4, 5: It would be good to rewrite the figure legends to detail what is plotted on each panel. Also, it would be better to position figure labels (A), (B), (C), (D) above the corresponding panel, not below.

We substantially reworded the figure legends in the revised manuscript and added more detailed information. We follow the convention from papers for computational approaches or signal processing and place the labels below.

Figure 5: It would be good to rescale the Y axis to show the whole bar. Starting the bar from 0.4 is a bit misleading.

We agree that starting the bar plot from 0.4 (i.e. 40%) can appear unusual. We measure here the simple correlation of the Pearson clusters (representing similarity in nucleosome phasing) to two groups of another genomic property (in the following *genomic group*). We opted for a statistical approach in which the cluster (i.e. 1 or 2) needed to be predicted based on an input (i.e. genomic group). If there is no information in the input to predict the cluster, then the program would randomly guess the group. This is equivalent to tossing a fair coin, where the probability of correctly predicting head or tail is 50%. Therefore, the bar below 0.5 contains no information. To simplify visual interpretability, we removed this part from the plot.

Is Figure 5 showing model performance around 50% (60% in best cases)? I am a bit confused if it is so. A performance of a ML model with ~50% correct predictions means that the model's performance is comparable to random guessing, so this aspect needs to be a bit better explained.

It is absolutely correct that 50% is equivalent to random guessing when having only two classes to predict. We want to emphasise that we did not aim to find a predictive ML model. As we include deliberately no hidden neurons, this approach allowed us to measure direct correlation between similarity of nucleosome phasing and other genomics properties. We clarified this point in the revised version on page 13 (lines 396-398):

> *We want to stress that we did not aim to find a predictive model. Rather, this approach allowed us to measure a direct correlation between similarity of nucleosome phasing and other genomics properties.*

Methods section: in general, it needs to be upgraded to make the description self-sufficient to reproduce. More specific points about the Methods section are listed below:

Please describe in more detail all parameters, and if the calculation was done with in-house scripts, please include these scripts as supplementary materials or make them available online and provide a link.

We substantially revised the Methods section and added more information about each step, particularly including more equations. We apologise for not publishing the GitHub repository in time. We added the link together with a versioned DOI in the revised version.

Line 458: "…inter and intra Pearson correlation…". Unclear what this means.

We agree that this wording is not clear, and we did not re-use it in the revised version.

Please explain all abbreviations, e.g. "JS", "PI", etc.

All abbreviations are introduced with their full explanation when they are first used in the main text (for example JS in line 145 (line 115 in the previous version) and PI in line 147 (line 116 in the previous version)). We avoid re-introducing them in several parts.

Figure 7: several points about this figure are listed below:

As recommended by Reviewer #1, Figure 7 was merged with Figure 1 in the revised manuscript and completely removed from this part.

It's unclear what Figure 7A means. Please add proper captions on the X and Y axes, move the color-code legend away from the Y-axis and rescale it to avoid impression that it's related to the Y-axis caption and add more detailed description to the figure legend.

We agree that this figure did not help visualising the Pearson correlations, and we removed it from the revised version.

I suggest to make heatmaps as in Figures 7C and 7D, separately for small and for large genes, and include them in the main manuscript to support the main conclusions of the study.

This is a very good suggestion. We created the plots and added them to the revised version. However, we see them rather supplementary and added them to the appendix instead (SFig A3).

I suggest to indicate on the Y axis of each heatmap figure, how many genes is in each cluster. Please move the color-code legend away from the Y axis, otherwise it can be confused with the numbers of genes (the numbers of horizontal lines on the heatmap)

We try to improve these points in the revised version. We added the number of genes and removed the colour bar, as this gives little information about the data itself. Instead, we added an explainer to the legend.

"A decrease of nucleosome as a function of distance". Should it be "A decrease of nucleosome phasing as a function of distance"?

We thank Reviewer #2 for pointing this out. This phrase was removed when figures and captions were merged for the revised manuscript.

Lines 515-516: “As we consider only two replicates, we restrain from using the word significant as much as possible and use noteworthy or notable instead.” It would be still good to explain in the manuscript, which results are statistically significant and which are not.

We are unsure what Reviewer #2 is suggesting here, as this is exactly the point we are trying to make. As the difference between the two slopes of replicates A and B can be considerably large, it would be not correct to state that the tilt of the linear boundary changed significantly between WT and mutant strain. This is why we use *noteworthy* or *notable* instead. We used the word *significant* wherever appropriate (i.e. for the established Pearson clusters).

Reviewer #3: Zeitler et al. describe a novel method of comparing MNase-seq datasets in an unbiased manner. Using fPCA of MNase composite plots for every gene, they characterized global trends in MNase sensitivity in a variety of previously published MNase-seq datasets performed on chromatin remodeler-deletion yeast strains. Their primary conclusions include establishing the RSC complex as required for global nucleosome positioning and showing evidence for Chd1 being primarily responsible for local arrangements of nucleosome positioning at the start of gene bodies. The technique demonstrated (fPCA) is novel for this application and appears to work at a global level. However, the largest outstanding concern is not with the nature of the bioinformatic analysis itself, but at a biochemical level regarding whether the distinct MNase datasets are comparable at the global level.

Major Points:
• This paper reanalyzes MNase- seq data generated from two distinct publications (Ocampo 2019 and Ocampo 2016). However, it is unclear from the analysis performed within the paper whether the MNase datasets were fundamentally comparable in the context of this analysis. Slight technical changes in MNase concentration have previously been demonstrated to dramatically change the relative occupancy of nucleosomes across the gene body and changes in MNase digestion paired to specific size selection can enrich for variably phased nucleosomes (https://pubmed.ncbi.nlm.nih.gov/22559821/). It would be critical to demonstrate the datasets are comparable from an MNase digestion perspective. More generally, are there genomic regions across the deletion strains that do not change in nucleosome phasing or occupancy?
• It is unclear what the implications of this statement mean for their algorithm. Are these false-negative datasets or does this speak to variability in MNase digestion profile? "For a correct interpretation of the results, it is crucial to highlight that this does not imply that other mutants had no effect on the nucleosome profile. In fact, other gene deletions that were not marked as being significant resulted also in visibly altered MNase-seq signals."

This is a very good and important point. Ocampo et al. (2019) combined both data sets themselves in their publication, which gives us confidence that the experimental procedures were comparable. Both publications are from the same laboratory with the same list of authors and the same first author. In the Methods section of Ocampo et al. (2019), the reference for MNase digestion and overall MNase-seq experiments is exactly the publication from the same laboratory by Ocampo et al 2016.

> *Nucleosomal DNA was prepared by MNase digestion of isolated nuclei and subjected to paired-end sequencing (Cole et al. 2011, 2012). These experiments,*

including biological replicates, were performed as described (Ocampo et al. 2016).

Moreover, as we showed in Fig 3 for Reviewers, we can reproduce the results for *rsc8*-depleted cells for a completely independent *rsc8-ts* strain that was produced by our laboratory (André et al., 2023). This suggests that our method is robust and fairly independent of MNase concentrations, and the results should be valid.

Minor Points:
- Legend in Figure 1B, 2A, 2B, 2C, 3-all, 5B, 5D, 5F are obscuring data and full interpretation
- Same issue with scatterplots in supplementary figures

We agree that it is a shame that the legend overlaps with parts of the scatter plot. However, the information carried in these figures is the linear separability and the slope of the discriminating boundary, which is clearly visible in all plots. Since the legend is slightly transparent, it is clear that some of the points are present underneath, and we do not hide the data for any interpretation. We deem the information in the legends to be more important.

References

André, K. M., Giordanengo Aiach, N., Martinez-Fernandez, V., Zeitler, L., Alberti, A., Goldar, A., ... & Soutourina, J. (2023). Functional interplay between Mediator and RSC chromatin remodeling complex controls nucleosome-depleted region maintenance at promoters. Cell Reports, 42(5).

Baldi, S., Krebs, S., Blum, H., & Becker, P. B. (2018). Genome-wide measurement of local nucleosome array regularity and spacing by nanopore sequencing. Nature structural & molecular biology, 25(9), 894-901.

Cole, H. A., Howard, B. H., & Clark, D. J. (2011). Activation-induced disruption of nucleosome position clusters on the coding regions of Gcn4-dependent genes extends into neighbouring genes. Nucleic acids research, 39(22), 9521-9535.

Koerber, R. T., Rhee, H. S., Jiang, C., & Pugh, B. F. (2009). Interaction of transcriptional regulators with specific nucleosomes across the Saccharomyces genome. Molecular cell, 35(6), 889-902.

Ocampo, J., Chereji, R. V., Eriksson, P. R., & Clark, D. J. (2016). The ISW1 and CHD1 ATP-dependent chromatin remodelers compete to set nucleosome spacing in vivo. Nucleic acids research, 44(10), 4625-4635.

Ocampo, J., Chereji, R.V., Eriksson, P.R., and Clark, D.J. (2019) Contrasting roles of the rsc and isw1/chd1 chromatin remodelers in rna polymerase ii elongation and termination. Genome research, 29(3):407–417.
