# Supplementary material for: A genome-wide comprehensive analysis of nucleosome positioning in yeast
Source: PLoS Comput Biol. 2024 Jan 24;20(1):e1011799. doi: 10.1371/journal.pcbi.1011799 (PMC10843174; doi:10.1371/journal.pcbi.1011799)
Supplement: Attachment — Submitted filename: Response to Reviewers 2.pdf [file pcbi.1011799.s018.pdf]

Response to Second Review for PLOS Computational Biology Submission PCOMPBIOL-D-23-01024R1: A Comprehensive Analysis of Nucleosome Positioning in Yeast

Introduction and general response to main concerns

Once again, we thank the reviewers for taking the time to provide valuable feedback to improve our manuscript and study. Before we provide a point-to-point response below, we want to briefly summarise our main changes in the revised manuscript.

One of the reviewers' main concerns was the comparison of the *k*-mean clustering with respect to other clustering approaches. We included a brief discussion of the results when using the WARD clustering algorithm on Euclidean distances (page 17 revised manuscript, SFig 14 revised manuscript). We also cite NucTools (Vainshtein et al., 2017) as an alternative tool that groups nucleosome profiles using *k*-mean clustering. Furthermore, we included the effect of other fPCs that describe less variance to improve the interpretability of the major two (SFig 3). Lastly, there was raised concern regarding our figures. We moved the legends and numeration in all plots. We adapted the colouring of the scatter plots such that the cluster that contains more dots in the top half is displayed in blue. Moreover, we re-phrased captions where required.

As in our previous response, the original remarks by the reviewers are given in black, whereas our reply is printed in red font.

Reviewer #1: Regarding the first point about explained variance in the principal component analysis on Page 17/115, the authors chose not to include the percentage of variance explained, reasoning that it's irrelevant to their analysis. While I understand the argument, I still believe a supplementary plot resembling Figure 1(F) would be beneficial. This plot should illustrate the variance explained by the top 10 functional Principal Components (fPCs), followed by a discussion on why the values are low. The lower explained variance might indicate limitations in the model's ability to capture the complexity of the data.

We would like to point Reviewer 1's attention to Page 6/ Line 211 of the previous version, where we do disclose the explained variance by the first two fPCs, and we cite directly:

> Despite the fact that the ratio of explained variance is not high (21.4% and 11.5% for fPC1 and fPC2, respectively), they are completely sufficient to distinguish between the Pearson correlation groups and permit an interpretation of the linear separating boundary between the clusters.

Before providing a brief discussion about the low explained variance, we want to emphasise that we never denied that the MNase-seq data themselves are complex. However, the first two fPCs are sufficient to linearly separate the Pearson clusters. Consequently, *they capture almost the entire variance measured by linear correlation.* Reciprocally, *Pearson correlation alone describes a considerable fraction of the variance* (which is captured by the first two fPCs). We opted particularly for Pearson clustering as it is similar to previously used indices such as autocorrelation (Wan et al., 2009) and a combination of Pearson correlation with coverage (Deniz et al., 2016). Pearson correlation was similarly previously used to compare

nucleosome positioning along genes before and after replication (Vasseur et al., 2016). In our study, we show what is captured by these measurements and how to interpret them.

Following Reviewer 1's suggestion, we added a figure to the Supplementary Data (SFig 3 in the revised version), and we included a discussion in the figure caption. We write the following:

> The first two fPCs are the ones that were presented in Fig 1(F) (21.3% and 11.5% explained variance, respectively). The fPCs that follow after the major two ones become increasingly complex, and it is difficult to quantify their effect in a straightforward measurement. With the exception of fPC4 (7.8% explained variance), the plots suggest that the effects of the fPCs that were not included would not have been captured by the linear correlation index, as they describe changes specific to a single nucleosome (e.g. fPC6) or complex changes (e.g. fPC7). We want to remind that the variance captured by the fPCs could either moderately occur along the majority of genes; or alternatively, there is a strong effect on a small subset of profiles. We interpret the results as follows. FPC1 and fPC2 show global trends how nucleosome arrangements change along most protein-coding regions. This is emphasised by the fact that they capture the most deviance from the mean and that their effects are not specific to particular nucleosome positions. Many other fPCs (e.g fPCs 5, 6, and 7, 7.5%, 7%, and 6.1% explained variance, respectively) include strong position-specific effects. Despite the large impact on the amplitude at precise positions (e.g. +3 or +4), the explained variance by these fPCs is lower. We presume that this indicates a strong variance at these positions in a small subset of genes. Thus, they show a much lower variance. We focus on the first two fPCs because they can separate the Pearson clusters, which in turn indicates that they capture global trends since they were determined over all genes.

The reason why we deem the explained variance to be of less importance is because they heavily depend on the number of basis functions used. In our study, we used 20 BSplines, and the explained variance can only be understood with respect to the 20 BSplines we used to represent the data. A link to the initial data complexity (i.e. 1200 bp) is not directly possible. We wanted to emphasise this point by showing the Pareto plot for 10 and 20 BSplines (Fig 1 for Reviewers). When using 10 BSplines, only six fPCs are necessary to explain 80%, whereas ten are required when using 20. This is similarly reflected by the fact that when using 10 BSplines, the first two fPCs explain almost 50% of the variance, whilst it is only approximately 33% when using 20 BSplines. We showed in our previous Response to Reviewers that the Pearson clusters are also largely linearly separable when using 10 BSplines.

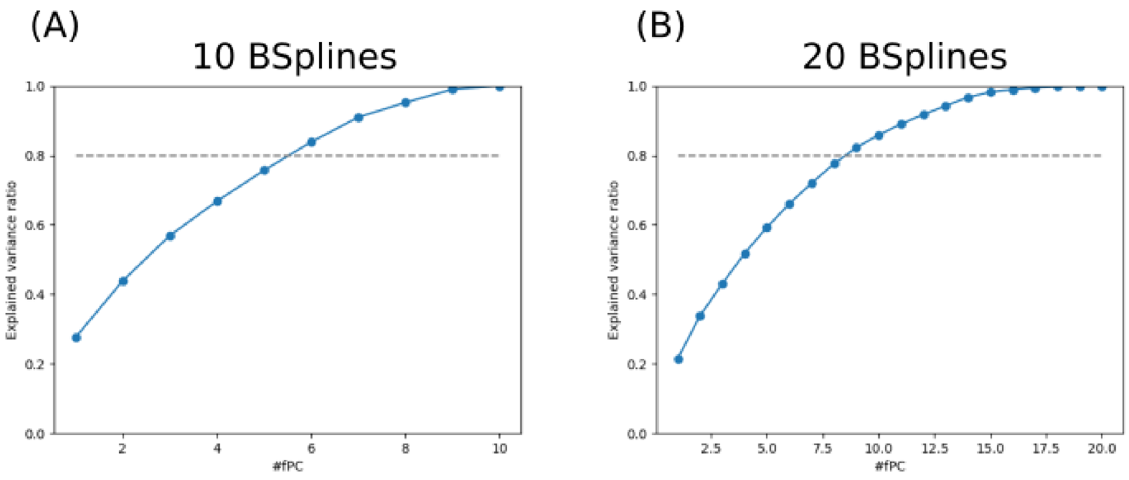

Fig 1 for Reviewers. The figure shows the explained variance over the number of fPCs using a different number of BSplines. (A) displays the Pareto plot using 10 BSplines, and (B) gives the explained variance using 20 BSplines. It is clear that the explained variance ratio is strongly dependent on the initial number of base functions used. Using this metric in the context of the raw data complexity is therefore not straightforwardly possible.

Concerning the methodology on Pages 5/115 and 17/115, the authors have not yet fully addressed questions regarding the suitability of Pearson correlation and k-means clustering. To enhance the rigor of the study, it would be instructive for the authors to explore alternative correlation methods and clustering algorithms to verify that their findings are not artifacts of the selected methodology. If Pearson correlation and k-means clustering are particularly well-suited to this problem, a detailed justification would be appreciated.

As mentioned by Reviewer 2, the *k*-means clustering algorithm has been previously applied to MNase-seq data, which showcased its suitability (Vainshtein et al., 2017). Nonetheless, we agree with Reviewer 1 that this would increase the study rigour, and we included the results of an alternative clustering approach in our manuscript (Discussion section page 17, lines 479-503, and SFig 14 in the revised version). Here, we want to briefly summarise the approach and results.

We repeated the clustering on the MNase-seq signal over protein-coding regions using agglomerative WARD clustering with an Euclidean distance metric instead of the Pearson correlation. Contrary to the Pearson correlation, the Euclidean distance does not have an upper bound, and the results were more sensitive to outliers. We removed 29 values that were outside the [-20, 20] range for any of the fPC scores for the first two fPCs. Once again, we can show that the silhouette criterion is largest when having two clusters, although the values are lower than for the Pearson clusters (SFig 14 (A) in the revised version). When plotting the cluster distribution with respect to the fPC scores, we find again that the two clusters tend to be grouped together in similar areas, although not as neatly as when using the *k*-mean clustering on the Pearson correlation indices (SFig 14 (B) in the revised version).

Moreover, the separation tends now to occur with respect to the first fPC rather than the second. This indicates that the Euclidean distance metric attributes a larger importance to the signal amplitude when combined with WARD clustering. This outcome emphasises the

following three points. Firstly, when dividing the set of protein-coding genes with respect to their nucleosome profiles, they can be best grouped into two clusters, and this is not limited to the choice of the *k*-mean or the Pearson correlation metric. Secondly, when including the scaling of the profile amplitude, profiles tend to be clustered into high and low presence (as suggested by the separability along fPC1). Therefore, the clustering does not express the similarity of the overall nucleosome positioning along the entire array. Thirdly, despite the fact that differentiation between the gene groups is based on other properties when applying WARD clustering, the first two fPCs carry a sufficient amount of information to similarly allow a separation, and it is hence not restricted to the Pearson clusters in our study. As indicated by the silhouette criterion value, the boundary is less clear, which speaks for our argument that genes can be better clustered with respect to their linear correlation. We want to emphasise that we were particularly interested in how the entire nucleosome array tends to behave as a *whole*, which should be sensibly measured by using the Pearson correlation. We decided to ignore the scaling of the sequencing amplitude, as this only conveys how many cells contain a single well-positioned nucleosome. We find the choice of *k*-mean clustering on Pearson indices for our study reasonable.

For Figure 1B and C, it would be helpful if the authors could clarify the choice of the color scale for the heatmaps. Is the chosen color scale perceptually uniform? On Line 153, the phrase “a different description of it” is a bit vague; some clarification would be beneficial. Similarly, at Line 207, given that a majority (~73%) of all genes in the study are large, the authors should discuss the potential bias in the findings regarding gene similarity.

We added the following sentences to the legend of Fig 1 in the manuscript:

> (B) and (C) display the profiles for each cluster. Large values are given in copper, low values are black. Both heatmaps are normalised independently, such that their respective largest value is displayed in the strongest copper hue and their lowest value in black.

We only found the sub-phrase “a different description of it” in Line 171 in the previous manuscript version (not in line 153). We changed the phrasing as follows:

> Instead, it is possible to investigate how the clusters distribute with respect to the data itself; or, alternatively, with respect to a different description using dimensionality reduction methods.

It is not clear to us what Reviewer 1 means by “the authors should discuss the potential bias in the findings regarding gene similarity”, as the entire Section “FPCA Reveals Size-Dependent Rsc8-Mediated Phasing of Nucleosome Positions” is dedicated to assessing the effect of different gene sizes (small < 1000, large between 1000 and 3500, and very large >3500) on clustering and fPCA and how they influence the observed results. With respect to small genes, this is summarised in Fig 2 (both manuscript versions). After this section, we exclusively use large genes that make up the majority of the considered protein-coding regions (i.e. 73%).

With respect to Figure 2, the authors mention in the legend for panels D and F that the wave-like pattern in the second functional Principal Component (fPC) dissipates after the +2 nucleosomes. However, this doesn't seem to align with the data presented. I would like the

authors to clarify this discrepancy. Also, it would improve readability if the legend information for panels A-C and D-F were grouped together.

We followed Reviewer 1's suggestions and clarified our figure legend as follows:

> [...] (D) The effect of two fPCs sheds light on why the Pearson groups are not linearly separable in WT using small-gene fPCs. The distribution of the second fPC changes its regular wave-like form to much broader peaks and valleys after the +2 nucleosome, which corresponds to approximately the size of the smallest genes in budding yeast. (E) Nucleosome positioning in *rsc8*-depleted conditions is clearly visible along the entire considered region, despite the included genes being smaller. This suggests that gene-specific nucleosome arrangement cannot be maintained. It is of note that the phasing also changes for the +1 nucleosome, and the NDR can be seemingly not conserved. (F) On the other hand, *rsc8*-depleted *chd1Δ* loses the regular wave-like shape of its second fPC after the +2 nucleosome to form broader peaks, indicating the presence of gene-specific nucleosome profiles as in WT conditions. [...]

We hope that this clarification addresses Reviewer 1's concerns.

Lastly, the issue regarding the isw1 deletion mutant deserves more attention. The mutant potentially affects both Isw1a and Isw1b complexes, which have distinct functionalities according to Yen et al., 2012. I would like the authors to consider whether the observed effects from +2 nucleosomes onwards could be attributed to Isw1b and provide their thoughts on this matter.

We misunderstood this point in the previous review, as we thought Reviewer 1 was referring to the two sequencing replicates for the *isw1*∆ strains instead of the two complexes, Isw1a and b. We agree with Reviewer 1 that Isw1 deletion can affect both complexes. Indeed, Isw1 is a common subunit of Isw1a and Isw1b complexes. Previous publications suggest that the two complexes are not equivalent in their functions and have different enrichment profiles: Ioc3 Isw1a subunit is enriched at +1 nucleosome, and Ioc4 Isw1b subunit is enriched on +2, +3, +4 nucleosomes. In addition, two complexes can act on distinct sets of genes and promote sliding in different directions. In this work, we did not analyse the effect of specific subunits of Isw1a or Isw1b complexes, so we cannot distinguish between the two complexes.

Reviewer #2: The authors have addressed most of my points, the method is well explained and the manuscript is now much clearer. The only point from my initial review that still needs a bit of attention is this one:

"Figure 2: Panels A-C here seem to report the main finding of the disappearance/reappearance of the cluster separation. Please try to reproduce the effect of disappearance/reappearance of the cluster separation using some other method (e.g. perform k-means clustering with one of standard software packages that exist for classical MNase-seq analysis), or explain why it can not be reproduced using another method. If it can not be reproduced with other methods it is really important to explain what these clusters represent, both mathematically and biologically. Are they characterised by different averaged nucleosome profiles? Then it would be good to show such different averaged profiles in the

manuscript."

To clarify, I suggest a couple of very small action points here:
1) The authors can at least mention NucTools that offers a similar functionality of k-means clustering of MNase-seq (https://bmcgenomics.biomedcentral.com/articles/10.1186/s12864-017-3580-2).

We have added NucTools as a possible alternative clustering software in our manuscript (Line 489 in the revised version). However, we were unable to use the CMBT package due to its compatibility issues with the operating system and the unavailability of the required MATLAB version licence before the response deadline. We hope our discussion using the WARD clustering algorithm with an Euclidean distance metric, which we included in our manuscript as detailed in our response to Reviewer 1, sufficiently addresses Reviewer 2's concern.

2) It would be still good to clarify, what is the biological and mathematical meaning of these clusters. I suggested initially to show the averaged nucleosome profiles for each cluster. I meant the aggregate profiles of the nucleosome occupancy for each cluster (not the principal components, but the actual nucleosome occupancy), and then discuss briefly the biological meaning of these profiles. Some hint to such biological interpretation is already provided in the schematic figure 1D, and it would be good to support it by actual nucleosome occupancy profiles.

We provide the average profiles in Fig 2 for Reviewers. Similar to what we described in our manuscript using Fig 3(C), there is particularly a difference in trend and shift after the +2 nucleosome. Moreover, occupancy levels tend to increase as a function of distance from the TSS for cluster 1, whereas they decrease for cluster 2. This indicates that the presence of nucleosomes closer to the TSS could affect presence farther into the gene body (and/or vice versa), and it therefore suggests how the nucleosome array behaves as a whole. These results are almost identical to what we presented in our study using fPCA, and we cite from our manuscript (line 205-207 in the previous version, line 212-214 in the revised version):

> By analysing the effect of the second fPC on the function shape, we conclude that the clusters are determined based on the downstream presence of nucleosomes (corresponding to the right cartoon in Fig 1(D)).

Regarding Reviewer 2's comment to "discuss briefly the biological meaning of these profiles", we are uncertain about what they require us to improve, as the entire Section Pol II Presence Correlates With Nucleosome Organisation in *chd1*Δ Mutants is dedicated to finding this out (for WT and different mutants). To summarise these findings for WT, replicates A and B show different correlations between the clusters and other genomic factors (such as Pol II presence or AT content). Moreover, any indicated correlation is only weak, which does not allow us to make strong conclusions about the biological meaning of the two clusters in WT. However, we find it remarkable that a link to Pol II occupancy is not as clear with the data that we have at hand as it has been suggested by other findings (e.g. Singh et al., 2021).

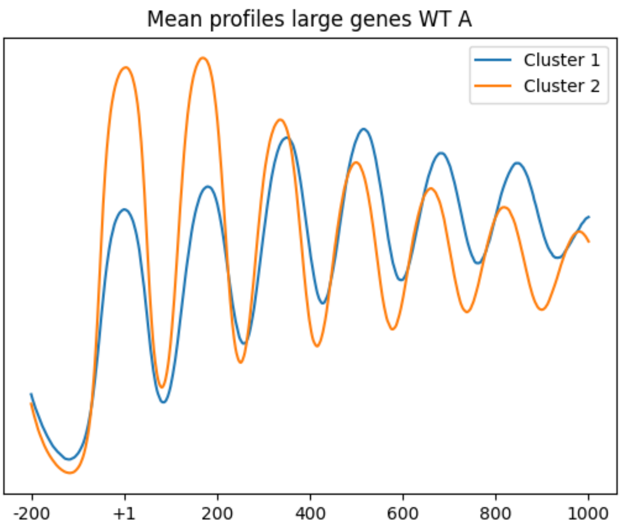

Fig 2 for Reviewers. The figure shows the average nucleosome profiles for clusters 1 and 2 on the raw MNase-seq data in WT. Signal amplitude tends to decrease for cluster 2, whereas it increases for cluster 1. This is exactly the phenomenon that is largely described by fPC2, which indicates that the method correctly captures and represents the trends observed in the data.

Reviewer #3: The authors have responded to my concern regarding MNase digestion bias. However, they notably have declined to address any of the other issues.

We are sad to hear that Reviewer 3 adopts the opinion that we did not address their other concerns. In their previous comments, they were particularly disquiet regarding the samples we did not include in our analysis. Throughout our study, we opted to be cautious and considered only observations that were measurable using our methodology and assumptions. We are convinced that including replicates that exhibit a sizable dissimilarity is irresponsible in an assessment that characterises variance in nucleosome position.

We want to explain this point a bit further. We use the data published by Ocampo et al. in 2016 and 2019 as ground truth. The MNase-seq signal over each gene was normalised to zero mean and standard deviation of one. We assessed the similarity between the two replicates before engaging in any analysis using the Kolmogorov-Smirnov (KS) test over 1000 randomly sampled values for each gene (see Fig 3 for Reviewers). Only 3% fulfil the null hypothesis that both replicates follow the same distribution (p-value 95% or higher, meaning that the probability that the two profiles follow a different distribution is 5% or lower). However, most deviance between the two replicates affects predominantly peak widths but not peak positions. We concluded that the mean collective behaviour remains largely preserved between the replicates. This was assessed using a methodology widespread in spectroscopy for comparing spectral patterns. For each position along a nucleosome profile, we determined the pairwise difference in z-scores between samples (K value). The distribution over all K values approaches a Voigt distribution, rather than a Gaussian distribution (see Fig 4 for Reviewers). As the Voigt distribution corresponds to the convolution between a Gaussian, a Lorentz, and a Dirac distribution, it suggests that the two considered replicates represent the same phenomena (given by the Dirac distribution).

However, because of the different sequencing depth, the base of the K values widens (included by the convolution with the Gaussian distribution) with a constraint (. This is also reflected by the fact that the two clusters similarly distribute along the first two fPCs between the replicates. Nonetheless, it is intuitive that a strong deviance will at some point influence a variance-based analysis approach such as fPCA or conventional PCA. In order to quantify this impact, we introduced the significance test in Eq 7 and explained its interpretation in Eq 8 of the revised manuscript. Based on this index, we removed strains for which the variability between the replicates exceeds the measurable change in slope with respect to the WT. In other words, the deviance of the first two fPCs between the replicates is larger than the difference of the average between the considered strain and WT. When keeping in mind that in the WT strain only 3% fulfil the null hypothesis for the same distribution, we find it reasonable to remove strains for which fPCA is more sensitive.

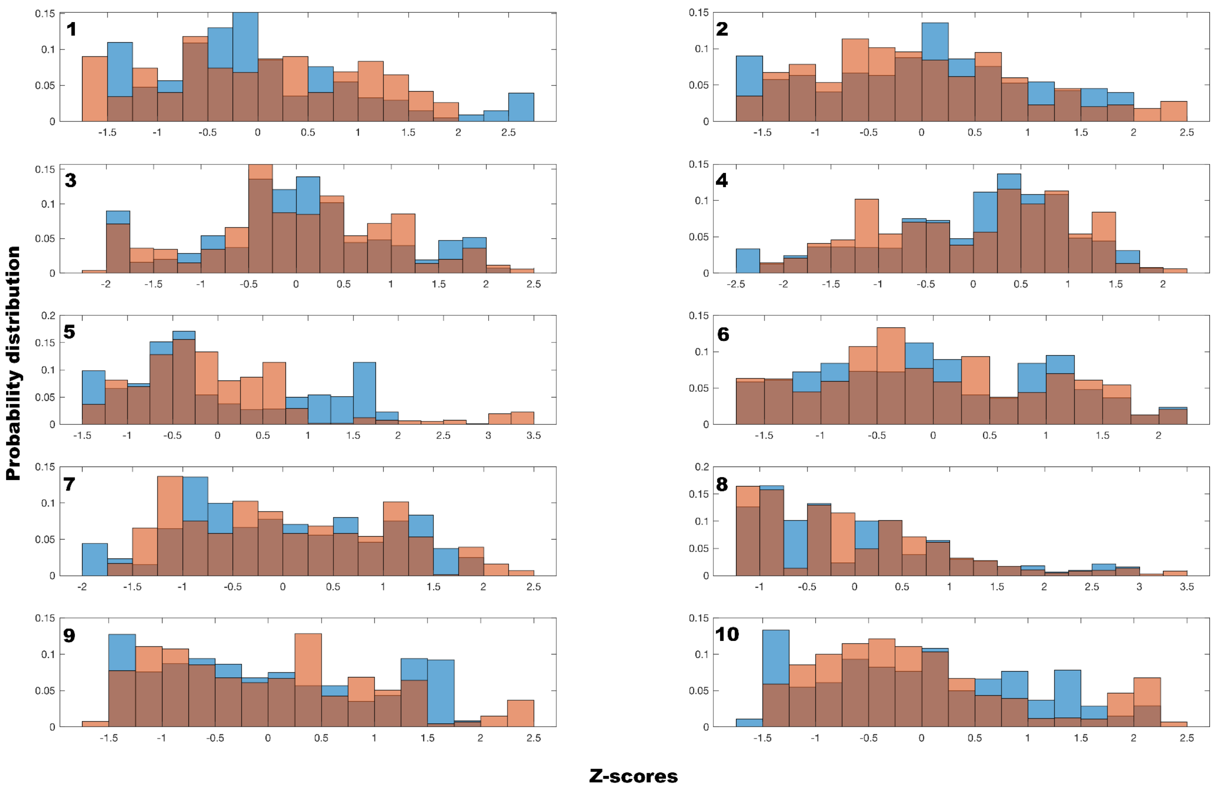

Fig 3 for Reviewers. The figure shows the z-score distribution for ten arbitrarily chosen WT genes. The blue histogram displays replicate A, and the orange bars show replicate B. A KS significance test between both replicates for each gene pair concluded that the z-score distributions are not significantly similar, with the exception of gene #6.

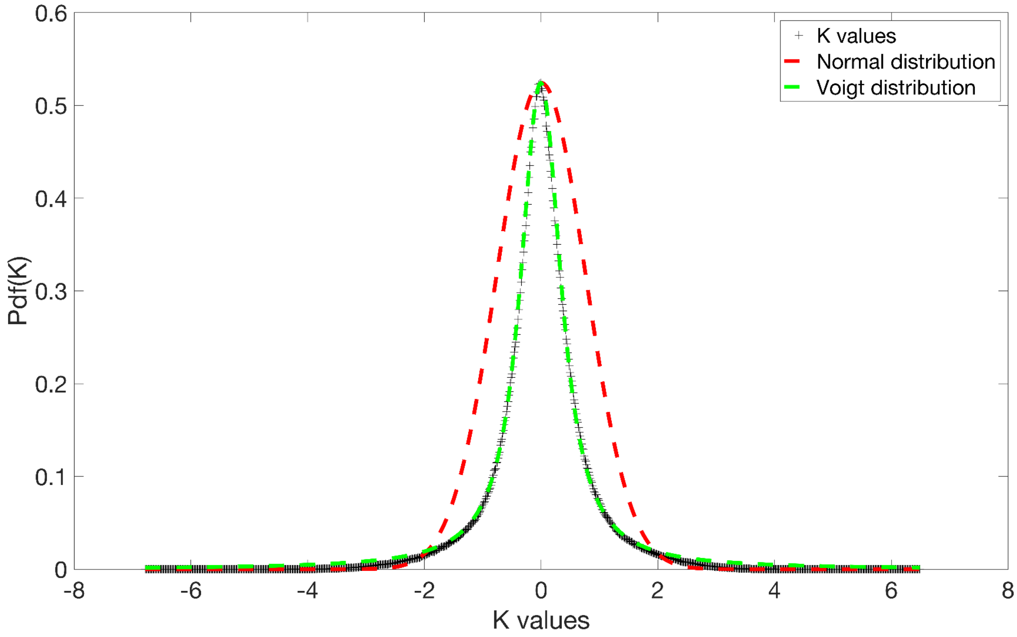

Fig 4 for Reviewers. The figure shows the pairwise z-score difference (K values) distribution for all WT genes in the two replicates A and B (grey plus symbols). The red dashed line corresponds to the best fit to the distribution of K values by a Normal distribution (mean=0, sigma = 0.76). The green dashed line corresponds to the best fit by a Voigt distribution (mean=0, gamma = 0.37, sigma = 0.11).

In the following paragraphs, we will elaborate on our decision with other wordings than those of the previous response.

My original major concern still stands which is: what is the false-negative interpretation rate of their approach? The authors modified the text I referenced in the original manuscript, but the replacement text is equally unclear. If the authors find visual differences in the chromatin remodeler deletion strains but their fPCA implementation does not identify them as significant, is this not a critical issue in their approach? What is meant by a 'gene-specific' variance and why would their approach not identify this?

Finally, we want to re-emphasize that purposefully providing occluded figures throughout the manuscript is not 'a shame' as the authors mention, but bad science. There is no justification for not moving figure legends outside the chart area.

We thank Reviewer 3 for clarifying their points. We want to first summarise our response in technical terms before giving an intuitive example. We previously tried to reply to their original major concern by re-explaining our measurement that quantifies changes in the slope by taking into account the variability between replicates. This approach allows us to select strains where the description of the Pearson clustering changed notably to the WT strain with respect to their linear separability by their first two fPCs. To rephrase what is described by the formula, there are two possibilities why we did not include a strain for a further downstream analysis. The first reason is rather intuitive because the variability between the two replicates was, for some strains, surprisingly large, and they did not allow

for real scientific conclusions (compare SFig 10 with SFig 11 in the revised version). The second possibility is that the linear correlation clusters were separated by the data variance similar to the WT strain. Before we elaborate on this point further by giving a concrete but arbitrary example (which we included in our revised manuscript SFig 12), please note that it was also possible to observe a combination of these two reasons.
Let us assume that some gene deletion caused the total depletion of the +2 nucleosome of large genes (i.e. > 1000) whilst leaving the position, fuzziness, and amplitude of all other nucleosomes in the array the same (SFig 12(A)). Naturally, this has a drastic effect on the mean, as there is no peak at the +2 position (SFig 12(B)). However, because of our study design, (a) this would not strongly influence the Pearson clusters as this change is neither a shift nor a general trend for which genes can be clustered into two groups; and (b) this would only weakly be represented by the two major fPC, since the total depletion along all large protein-coding regions would only induce some random and uninformative noise instead of affecting or inducing some major variation among the genes. Please note that the way how the array is organised as a whole with respect to the WT does not significantly change. Consequently, the separation between the two Pearson correlation clusters by the first two fPCs only changes slightly (SFig 12(C)). This influence is smaller than the variation between the two biological replicates, despite the fact that we completely replaced one entire nucleosome by random noise.

As repeatedly emphasised several times throughout the manuscript, as well as the two Responses to Reviewers, we aim to understand what is represented by the two Pearson clusters using fPCA, which we see as a surrogate for the organisation of the *entire* nucleosome array. Therefore, we are particularly interested in strains where this linear separability notably changes with respect to WT cells. Chromatin remodeler deficiencies that affect only the mean are of less interest to us. We never aimed (or claimed to be aiming) to quantify or visualise every single change that was induced through a gene deletion or protein depletion. Nevertheless, we acknowledge that other chromatin remodeler deletions can have various impacts, which might be different to the given example. We use the measurement (which we explained in detail to Reviewer 3 when addressing their original major concern) in order to specifically select strains that are within our study interest and can be sensibly represented with the used methods. We want to stress that there is nothing alien about our example given in this response, as we apply exclusively notions that are used in classic data analysis approaches. The same holds true for conventional PCA. We also find the use of fairly strong formulations like *a false-negative interpretation* or an *issue with our approach* slightly surprising, as it is common practice to filter the data to allow interpretability using a particular methodology. To avoid this misunderstanding with future readers, we added this example to the supplementary data in our manuscript (SFig 12).

Lastly, we would like to comment on Reviewer 3’s issue concerning the figures. Initially, we believed that having a transparent legend in the main figures was visually appealing. We thought the transparency would allow those who want to look at the figure to understand its purpose, as data points were still visible. Apparently, we were mistaken. We naturally changed the plots and moved the figure legends outside the main plot as well as the numeration above the plot.

Additional Modifications

We wrote in the Methods section that the clustering was performed on the Pearson correlation matrix itself (line 630-632 in the previous version). In the revised version, we specified that we applied the MATLAB k-mean clustering function directly on the profiles using the Pearson correlation as a distance metric (i.e. parameter Distance was set to correlation, line 658-659 in the revised manuscript). We also clarified this point in the Result section Nucleosome Profiles Can Be Well Distinguished Based On Their Coordinated Positioning in WT, and we write in lines 140-145 of the revised manuscript

> These Pearson coefficients were used as a distance metric to cluster nucleosome profiles into distinct partitions using *k*-mean clustering. In a nutshell, the algorithm divides a data set of *m* observations (the MNase-seq data) into *k* groups by minimising the variance within each cluster based on a distance metric (here, the pairwise Pearson indices over all genes). Therefore, genes within a group tend to have nucleosomes at comparable positions, whereas profiles of different groups are likely to be less similar.

We improved the significance test that was applied in order to validate the significance of the found Pearson clusters per mutant. Before, we used a Jensen-Shannon distance between all Pearson correlations of clusters 1 and 2, which we claimed to be as dissimilar as possible. However, this incorrectly included correlation indices to other profiles in the same cluster. This could become problematic when having, for example, two clusters that are perfectly symmetric yet are perfectly distinct (e.g. shifting one entire group by a constant bias). We adapted and improved the significance measurement as follows. We expect that when considering all pairwise gene correlations in different clusters (i.e. one profile is from cluster 1, whereas the other is from cluster 2), the distribution of these Pearson indices should be significantly lower than for random partitioning. This is now validated with a Kolmogorov-Smirnov (KS) test. We named the correlation values for profiles from different gene sets inter-cluster correlation. As the KS test can be very sensitive if sample sizes are large, we randomly sub-sample 500 inter-cluster indices for both the random and the *k*-mean Pearson clusters. To account for the random fluctuations in the sampling, we repeated the KS test over 500 clusters (with subsequent sub-sampling) and determined the average p-value. Using this approach, we can affirm that the clusters for large genes of all mutants are significant (i.e. average p-value < 5%), including the previously discarded *rsc8*-depleted *isw1Δ* replicate B. However, we want to stress that this did not jeopardise any major results of our study, as the *rsc8*-depleted *isw1Δ* mutant was not included in our downstream analysis. We explain our adapted approach in our revised manuscript version in the Results section lines 150-154 Nucleosome Profiles Can Be Well Distinguished Based On Their Coordinated Positioning in WT as well as in the Discussion section lines 505-515 and Methods section lines 664-676. We also visualise the differences between random and Pearson clusters in SFig 9.

Lastly, we realised that we incorrectly cited Chereji and Clark, 2018, for having used the Pearson correlation to compare nucleosome profiles directly. We corrected this in our revised manuscript version and added other studies that used Pearson correlation in similar setups or that used related measurements. We write in lines 78-81 (where we replaced the numbers with the actual references):

> [...] However, many of them rely predominantly on measurements that describe only an average over the entire profile, such as autocorrelation measurements (Wan et al, 2009) or Pearson correlation that was adapted to include coverage (Deniz et al, 2016). Pearson correlation was also used to compare nucleosome positioning of genes before and after replication (Vasseur et al., 2016). [...]

This citation was removed from all references where we mention Pearson correlation measurements of nucleosome profiles.

References

Chereji, R.V. and Clark, D.J., 2018. Major determinants of nucleosome positioning. *Biophysical journal*, *114*(10), pp.2279-2289.

Deniz, Ö., Flores, O., Aldea, M., Soler-López, M. and Orozco, M., 2016. Nucleosome architecture throughout the cell cycle. *Scientific reports*, *6*(1), pp.1-11.

Singh, A.K., Schauer, T., Pfaller, L., Straub, T. and Mueller-Planitz, F., 2021. The biogenesis and function of nucleosome arrays. *Nature communications*, *12*(1), p.7011.

Vainshtein, Y., Rippe, K. and Teif, V.B., 2017. NucTools: analysis of chromatin feature occupancy profiles from high-throughput sequencing data. *BMC genomics*, *18*, pp.1-13.

Vasseur, P., Tonazzini, S., Ziane, R., Camasses, A., Rando, O.J. and Radman-Livaja, M., 2016. Dynamics of nucleosome positioning maturation following genomic replication. *Cell reports*, *16*(10), pp.2651-2665.

Wan, J., Lin, J., Zack, D.J. and Qian, J., 2009. Relating periodicity of nucleosome organization and gene regulation. *Bioinformatics*, *25*(14), pp.1782-1788.
