# Supplementary material for: A genome-wide comprehensive analysis of nucleosome positioning in yeast
Source: PLoS Comput Biol. 2024 Jan 24;20(1):e1011799. doi: 10.1371/journal.pcbi.1011799 (PMC10843174; doi:10.1371/journal.pcbi.1011799)
Supplement: Attachment — Submitted filename: point-by-point-response.pdf [file pcbi.1011799.s020.pdf]

Response to Third Review for Submission to PLOS Computational Biology
PCOMPBIOL-D-23-01024R2: **A Comprehensive Analysis of Nucleosome Positioning in Yeast**

General Reply
We would like to thank the editors and the reviewers for their time and feedback. We highly appreciate their commitment, and we find that their comments helped us to substantially improve our work. In the present version, we addressed some minor concerns raised by Reviewer #1 by adding two additional paragraphs to the discussion. We also updated our GitHub repository according to the changes that we made. In the following, we provide a short point-by-point response to the remaining comments. As in our previous responses, we mark our replies in red font, whereas the original remarks are coloured in black.

The response clarifies the normalization of the heatmaps, with the largest values displayed in the strongest copper hue and the lowest values in black. However, the explanation does not address whether the color scale is perceptually uniform. Perceptual uniformity is crucial for accurately reflecting differences in the data through visual representation.

We added the following modification to the caption of Fig 1:

> [...] (B) and (C) display the profiles for each cluster. Large values are given in copper, low values are black, and the colour gradient in between is uniformly scaled. It is therefore a perceptually uniform representation.

In their analysis, the authors exclusively focus on large genes, which constitute the majority of the protein-coding regions studied. This selection might introduce bias, especially if conclusions drawn from large genes are generalized to the entire genome. The latter includes shorter genes, which, while they comprise only about 27% of the genome, are nonetheless significant. To enhance the robustness of their study, the authors should discuss how this focus on large genes may affect the overall applicability of their results. Moreover, they should outline any measures taken to mitigate potential bias or clarify that their conclusions are applicable primarily to large genes.

We included a paragraph to the Discussion section (page 19 in the revised manuscript), which clarifies the limitation to large genes:

> The length of an average yeast gene is approximately 1500 bp, which corresponds to 6-7 nucleosomes. To analyse the effect of nucleosome remodeler deletions or their depletion on their arrangement, we examined a stretch of 1200 bp per gene, including 200 bp upstream of the +1 dyad. By considering an array of 6-7 nucleosomes at the same time, we were able to assess their long-range impact on the gene body. During our analysis, we differentiated between small and large genes (i.e. coding regions shorter than the considered 1000 bp after the +1 dyad versus genes ≥1000 bp). This revealed the effect of Rsc8 on limiting nucleosome positioning to the gene body. It should be emphasised that we removed smaller genes from the subsequent analysis, as it would be impossible to compare nucleosome arrangement further down into the gene body. We also surmised that the influence of remodeler deletions on later nucleosomes is more difficult to

> observe in smaller genes, since they could be located very right at the TTS which could have an additional impact. Therefore, we based our analysis and conclusions—particularly with respect to Chd1—only on genes larger than 1000 bp. It should be noted that the results may not be easily transferable to small genes.

In light of the feedback regarding the isw1∆ deletion mutant, it appears that the distinction between the functionalities of the Isw1a and Isw1b complexes has not been explored in this study. Given that these complexes have distinct roles as reported in the literature, it would be valuable for the readers to understand that the observed effects from the +2 nucleosomes onwards may not be clearly attributable to one specific complex. We recommend that the manuscript explicitly acknowledge this limitation.

We commented on the constrained with respect to the remodeler complexes Isw1a and Isw1b in the Discussion section (page 20) with the following paragraph (the numbered citation was replaced):

> It is worth noting that the Isw1 subunit is part of the two complexes Isw1a and Isw1b, and consequently its deletion affects them both. Previous studies *(Yen et al., 2012)* have shown that these complexes have different functions and enrichment profiles. The Ioc3 subunit of the Isw1a complex is enriched at +1 nucleosome, while the (Isw1b) is enriched on +2, +3, and +4 nucleosomes. In addition, these two complexes can act on different sets of genes and promote sliding in different directions. However, as we did not analyse the effect of specific subunits of Isw1a or Isw1b complexes in this study, we cannot distinguish between the two complexes. Therefore, it is possible that the effects observed from the +2 nucleosome onward may not be due to one specific complex.

References

Yen, K., Vinayachandran, V., Batta, K., Koerber, R. T., & Pugh, B. F. (2012). Genome-wide nucleosome specificity and directionality of chromatin remodelers. *Cell*, *149*(7), 1461-1473.
