# Supplementary material for: A genome-wide comprehensive analysis of nucleosome positioning in yeast
Source: PLoS Comput Biol. 2024 Jan 24;20(1):e1011799. doi: 10.1371/journal.pcbi.1011799 (PMC10843174; doi:10.1371/journal.pcbi.1011799)
Supplement: Attachment — Submitted filename: response to rebuttal for submission 3 v2.docx [file pcbi.1011799.s019.docx]

- Regarding the first point about explained variance in the principal component analysis on Page 17/115, the authors chose not to include the percentage of variance explained, reasoning that it's irrelevant to their analysis. While I understand the argument, I still believe a supplementary plot resembling Figure 1(F) would be beneficial. This plot should illustrate the variance explained by the top 10 functional Principal Components (fPCs), followed by a discussion on why the values are low. The lower explained variance might indicate limitations in the model's ability to capture the complexity of the data.
- This reviewer is satisfied with the response.
- Concerning the methodology on Pages 5/115 and 17/115, the authors have not yet fully addressed questions regarding the suitability of Pearson correlation and k-means clustering. To enhance the rigor of the study, it would be instructive for the authors to explore alternative correlation methods and clustering algorithms to verify that their findings are not artifacts of the selected methodology. If Pearson correlation and k-means clustering are particularly well-suited to this problem, a detailed justification would be appreciated.

This reviewer is satisfied with the response.

- For Figure 1B and C, it would be helpful if the authors could clarify the choice of the color scale for the heatmaps. Is the chosen color scale perceptually uniform? On Line 153, the phrase “a different description of it” is a bit vague; some clarification would be beneficial. Similarly, at Line 207, given that a majority (~73%) of all genes in the study are large, the authors should discuss the potential bias in the findings regarding gene similarity.

The response clarifies the normalization of the heatmaps, with the largest values displayed in the strongest copper hue and the lowest values in black. However, the explanation does not address whether the color scale is perceptually uniform. Perceptual uniformity is crucial for accurately reflecting differences in the data through visual representation.

In their analysis, the authors exclusively focus on large genes, which constitute the majority of the protein-coding regions studied. This selection might introduce bias, especially if conclusions drawn from large genes are generalized to the entire genome. The latter includes shorter genes, which, while they comprise only about 27% of the genome, are nonetheless significant. To enhance the robustness of their study, the authors should discuss how this focus on large genes may affect the overall applicability of their results. Moreover, they should outline any measures taken to mitigate potential bias or clarify that their conclusions are applicable primarily to large genes.

- With respect to Figure 2, the authors mention in the legend for panels D and F that the wave-like pattern in the second functional Principal Component (fPC) dissipates after the +2 nucleosomes. However, this doesn't seem to align with the data presented. I would like the authors to clarify this discrepancy. Also, it would improve readability if the legend information for panels A-C and D-F were grouped together.

This reviewer is satisfied with the response.

- Lastly, the issue regarding the isw1 deletion mutant deserves more attention. The mutant potentially affects both Isw1a and Isw1b complexes, which have distinct functionalities according to Yen et al., 2012. I would like the authors to consider whether the observed effects from +2 nucleosomes onwards could be attributed to Isw1b and provide their thoughts on this matter.

In light of the feedback regarding the isw1∆ deletion mutant, it appears that the distinction between the functionalities of the Isw1a and Isw1b complexes has not been explored in this study. Given that these complexes have distinct roles as reported in the literature, it would be valuable for the readers to understand that the observed effects from the +2 nucleosomes onwards may not be clearly attributable to one specific complex. We recommend that the manuscript explicitly acknowledge this limitation.
